# High sodium intake, glomerular hyperfiltration, and protein catabolism in patients with essential hypertension

**DOI:** 10.1093/cvr/cvaa205

**Published:** 2020-07-16

**Authors:** Giacomo Rossitto, Giuseppe Maiolino, Silvia Lerco, Giulio Ceolotto, Gavin Blackburn, Sheon Mary, Giorgia Antonelli, Chiara Berton, Valeria Bisogni, Maurizio Cesari, Teresa Maria Seccia, Livia Lenzini, Alessio Pinato, Augusto Montezano, Rhian M Touyz, Mark C Petrie, Ronan Daly, Paul Welsh, Mario Plebani, Gian Paolo Rossi, Christian Delles

**Affiliations:** 1 Institute of Cardiovascular and Medical Sciences, BHF Glasgow Cardiovascular Research Centre 126 University Place, University of Glasgow, Glasgow G12 8TA, UK; 2 Clinica dell’Ipertensione, DIMED, University of Padua, University Hospital, via Giustiniani 2, Padua 35126, Italy; 3 Glasgow Polyomics, University of Glasgow, Wolfson Wohl Cancer Research Centre, Garscube Campus, Bearsden, Glasgow G61 1BD, UK; 4 Laboratory Medicine, DIMED, University of Padua, University Hospital, via Giustiniani 2, Padua 35126, Italy

**Keywords:** Salt, Hypertension, Kidney, Glomerular hyperfiltration, Metabolism

## Abstract

**Aims:**

A blood pressure (BP)-independent metabolic shift towards a catabolic state upon high sodium (Na^+^) diet, ultimately favouring body fluid preservation, has recently been described in pre-clinical controlled settings. We sought to investigate the real-life impact of high Na^+^ intake on measures of renal Na^+^/water handling and metabolic signatures, as surrogates for cardiovascular risk, in hypertensive patients.

**Methods and results:**

We analysed clinical and biochemical data from 766 consecutive patients with essential hypertension, collected at the time of screening for secondary causes. The systematic screening protocol included 24 h urine (24 h-u-) collection on usual diet and avoidance of renin–angiotensin–aldosterone system-confounding medications. Urinary 24 h-Na^+^ excretion, used to define classes of Na^+^ intake (low ≤2.3 g/day; medium 2.3–5 g/day; high >5 g/day), was an independent predictor of glomerular filtration rate after correction for age, sex, BP, BMI, aldosterone, and potassium excretion [*P* = 0.001; low: 94.1 (69.9–118.8) vs. high: 127.5 (108.3–147.8) mL/min/1.73 m^2^]. Renal Na^+^ and water handling diverged, with higher fractional excretion of Na^+^ and lower fractional excretion of water in those with evidence of high Na^+^ intake [FE_Na_: low 0.39% (0.30–0.47) vs. high 0.81% (0.73–0.98), *P* < 0.001; FE_water_: low 1.13% (0.73–1.72) vs. high 0.89% (0.69–1.12), *P* = 0.015]. Despite higher FE_Na_, these patients showed higher absolute 24 h Na^+^ reabsorption and higher associated tubular energy expenditure, estimated by tubular Na^+^/ATP stoichiometry, accordingly [Δhigh–low = 18 (12–24) kcal/day, *P* < 0.001]. At non-targeted liquid chromatography/mass spectrometry plasma metabolomics in an unselected subcohort (*n* = 67), metabolites which were more abundant in high versus low Na^+^ intake (*P* < 0.05) mostly entailed intermediates or end products of protein catabolism/urea cycle.

**Conclusion:**

When exposed to high Na^+^ intake, kidneys dissociate Na^+^ and water handling. In hypertensive patients, this comes at the cost of higher glomerular filtration rate, increased tubular energy expenditure, and protein catabolism from endogenous (muscle) or excess exogenous (dietary) sources. Glomerular hyperfiltration and the metabolic shift may have broad implications on global cardiovascular risk independent of BP.

## Graphical Abstract

**Figure:**
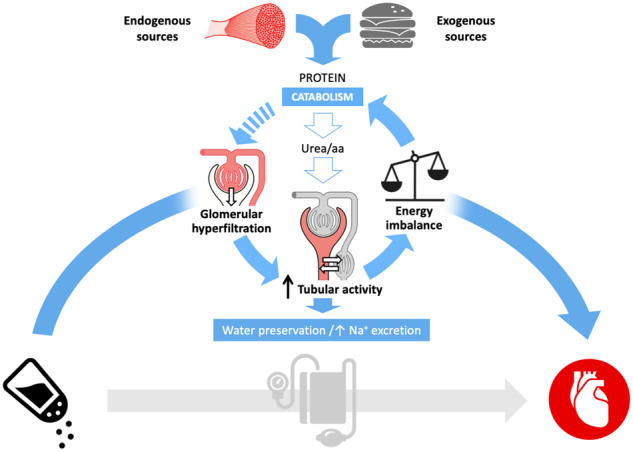

## 1. Introduction

A close association between sodium (Na^+^) and blood pressure (BP) is well recognized, although BP response to increased or reduced sodium intake (salt sensitivity) varies across populations and individuals.^1–4^ The rationale for World Health Organization reduced sodium intake recommendations is based on the predicted benefit in terms of incidence of cardiovascular events that would follow a population-level decrease in BP.^3^^,^^5^ While there is reasonable agreement that high salt intake (generally identified as > 5 g Na^+^/day) is associated with worse outcomes, there is no consensus on the optimal range of intake and the role for its effect on BP as the sole causal determinant of cardiovascular events has not been proved.^6–9^

Recently, an independent link between high Na^+^ intake and a shift in metabolism has been suggested, whereby a catabolic state induced by Na^+^ would ultimately serve preservation of body water. In particular, in the experimental setting of a long-term salt balance study in 10 healthy male subjects simulating a space flight, Rakova *et al.*^10^ demonstrated that endogenous water generation and accrual prevented extra water intake upon high salt diet. Further mechanistic insights from a rodent study pointed to urea excess as a key osmotic force to minimize water loss.^11^ This was achieved by renal urea recycling and extrarenal endogenous generation, via a salt-driven catabolic state. Based on evidence of increased 24 h-u-glucocorticoids excretion in both humans and mice, the authors suggested hypercortisolism to mediate this metabolic shift.^10^^,^^11^

These mechanisms, which would lead to increased cardiovascular risk regardless of BP salt-sensitivity, lack demonstration in humans out of a dietary- and environmentally controlled experimental setting.

This study sought to investigate the impact of high salt intake on renal Na^+^/water handling and metabolism in real-life in hypertensive patients undergoing systematic biochemical screening for secondary causes of hypertension following a pre-defined standardized protocol. We herein report the results for the large cohort of patients who received a final diagnosis of essential hypertension.

## 2. Methods

An expanded version is available in the Supplementary material online.

### 2.1 Patients and diagnostic protocol

The study included clinical and biochemical data from consecutive patients referred to the tertiary Hypertension Center of the University of Padua, who underwent a biochemical screening for secondary causes of hypertension and provided informed written consent (2012–17; local biobank Prot.1925P/2009; international ENSAT-HT network, http://www.ensat-ht.eu/, local approval prot. 3998/AO/16; in compliance with the Declaration of Helsinki). The screening entailed plasma electrolytes, aldosterone, renin, cortisol, 24 h-u-Na^+^, and K^+^ excretion; additional biochemical data, including renal function, 24 h creatinine excretion, 24 h urinary catecholamines/metanephrines, and urinary free cortisol were measured as recommended by guidelines.^12–15^ Patients were not instructed to change their usual dietary habits before the screening. They were asked to undertake a 24 h-u-collection on the day before and to bring the entire volume on the morning of blood sampling, which was performed after 1 h in supine position, between 8 and 10 a.m. If patients were already treated, screening was performed after appropriate washout from agents affecting the renin–angiotensin–aldosterone system and switch to calcium channels blockers and/or doxazosin, as per guidelines,^13^ unless contraindicated. Exclusion criteria entailed reported/biochemical evidence of oral, intramuscular or intravenous steroid use or abuse and cases with a final diagnosis of secondary hypertension after appropriate work-up (biochemistry, anatomical/functional imaging, adrenal/renal vein sampling), and follow-up at the time of data-lock (1 January 2019). Patients with a conclusive diagnosis of essential hypertension were grouped according to classes of Na^+^ intake. Intake estimates were based on 24 u-Na^+^-excretion, which has limitations when applied to a single subject^16^ but is only minimally affected by within-individual day-to-day variability when applied to groups with sufficient number of participants included.^17^ Intakes were defined as low ≤2.3 g Na^+^/day (100 mmol/day); medium 2.3–5 g Na^+^/day; high >5 g Na^+^/day (216 mmol/day; Supplementary material online, *Figure S1*), according to commonly used cut-offs.^18^^,^^19^

### 2.2 Laboratory methods

Plasma and urinary electrolytes, plasma renin, aldosterone and cortisol, and additional biochemistry as appropriate and detailed above, were measured at the time of the secondary hypertension screening in an International Standard Organisation (ISO) 15189:2012 accredited clinical laboratory (University of Padua) by routine methods. Normal values and within‐ and inter-assay coefficient of variation for aldosterone and renin, as well as aldosterone-to-renin ratio (ARR) criteria for primary aldosteronism and further diagnostic work-up have already been reported^13^^,^^20^^,^^21^ and are recapitulated in the Supplementary material online. Plasma cortisol concentration was measured by a chemiluminescence competitive immunoassay and urinary 24 h free cortisol (UFC) by a liquid chromatography–mass spectrometry (LC-MS)/MS, as described in detail elsewhere.^22^^,^^23^ Urinary catecholamines and metanephrines were measured by HPLC with electrochemical detection with a CE-IVD kit (see Supplementary material online).

Plasma and 24 h urinary urea and creatinine were not routinely determined in all screened patients; the available biochemical dataset was expanded by analysing urine samples stored in the local biobank since the time of the screening and validating the results against the original dataset according to Passing and Bablok method (Supplementary material online, *Figures S2–S4* and *Table S1*).

**Table 1:** Clinical and biochemical characteristics of patients, by Na^+^ intake group

	*n*_valid_	Whole cohort	Low-Na^+^ (*n* = 146)	Medium-Na^+^ (*n* = 464)	High-Na^+^ (*n* = 156)	*P*
Age (years)	766	47±13	47±13	47±13	44±13*,†	0.015
Sex (M; *n*/%)	766	428/55.9	49/33.6	248/53.4*	132/84.1*,†	<0.001
BMI (kg/m^2^)	537	25.6 (23.0–29.0)	24.1 (21.0–27.4)	25.7 (23.2–29.1)*	26.7 (24.5–29.8)*,†	<0.001
SBP (mmHg)	647	150±18	153±20	149±18	149±15	0.165
DBP (mmHg)	647	93±10	93±10	93±10	94±11	0.801
Medications						
None (*n*/%)	766	138/18.0	27/18.5	89/19.2	22/14.0	0.356
Dihydropiridine CCB (*n*/%)	766	444/58.0	82/56.2	261/56.3	102/65	0.158
Non-dihydropiridine CCB (*n*/%)	766	160/20.9	30/20.5	103/22.2	27/17.2	0.427
α-blockers (*n*/%)	766	173/22.6	24/16.4	102/22.0*	47/29.9*,†	0.016
Diabetes (*n*/%)	630	24/3.8	4/3.3	15/4.0	5/3.6	0.927
Chronic kidney disease (*n*/%)	645	24/3.7	4/3.3	18/4.7	2/1.4	0.222
p-Na^+^ (mmol/L)	675	141±2	141±2	141±2	141±2	0.650
p-K^+^ (mmol/L)	703	4.0±0.4	4.0±0.4	4.1±0.4	4.0±0.4	0.070
PAC (pmol/L)	766	241 (183–340)	265 ( 189–386)	232 (182–323)*	254 (174–344)*	0.040
PRA (ng/mL/h), 2012–15	313	0.64 (0.33–1.26)	1.00 (0.29–1.50)	0.61 (0.30–1.21)	0.62 (0.41–1.17)	0.361
DRC (mIU/L), 2015–17	452	7.9 (3.3–14.8)	9.5 (4.1–15.9)	7.7 (3.1–13.6)	7.6 (2.7–15.7)	0.155
ARR_PRA_ (ng/dl/ng/mL/h)	313	15.9 (9.1–29.4)	14.9 (9.1–29.7)	16.5 (8.2–31.2)	15.7 (9.8–24.7)	0.823
ARR_DRC_ (ng/dl/mIU/L)	452	1.09 (0.61–2.25)	0.99 (0.62–2.00)	1.14 (0.62–2.46)	1.03 (0.53–2.15)	0.514
24h-u-Diuresis (L/day)	766	1.8 ( 1.4–2.3)	1.5 (1.0–2.1)	1.8 (1.4–2.3)*	2.0 (1.6–2.4)*,†	<0.001
24h-u-Na^+^ (mmol/day)	766	155 (112–205)	80 (66–92)	154 (128–183)*	252 (236–294)*,†	<0.001
24h-u-K^+^ (mmol/day)	766	60 (48–77)	49 (38–65)	60 (48–74)*	73 (61–90)*,†	<0.001

*n*_valid_, number of patients with available information; BMI, body mass index; SBP and DBP, systolic and diastolic blood pressure, respectively; CCB, calcium channel blockers; p-, plasma; 24 h-u-, 24 h urine; PAC, plasma aldosterone concentration; PRA, plasma renin activity; DRC, direct renin concentration; ARR, aldosterone-to-renin ratio.

* *Post hoc* tests: *P* < 0.05 versus low-Na^+^; †*P* < 0.05 versus medium-Na^+^.

### 2.3 Renal function and energetics

Urine samples collected over the 24 h immediately before plasma sampling were used for estimation of glomerular and tubular function, according to standard equations. In particular, glomerular filtration rate (GFR) was estimated by 24 h creatinine clearance and also by the CKD-EPI formula.^24^ Tubular Na^+^ handling was assessed by fractional excretions (FEs) (i.e. the percentage of the filtered amount that is excreted), as well as the absolute amount of filtered, reabsorbed, and excreted Na^+^ (mmol/day). FE of water (FE_Water_) was calculated as urine flow rate/GFR. Estimated tubular energy expenditure (kcal/day) was calculated based on a tubular 4.6 Na^+^/ATP stoichiometry and the free energy equivalent of ATP.^25^

### 2.4 Metabolomics

Available EDTA-plasma samples from a non-selected sub-cohort of patients in the low and the high Na^+^ intake groups, stored in Padua biobank from the time of screening, were extracted with chloroform/methanol/water (1:3:1 v/v) and stored at −80°C until analysis by LC-MS. Briefly, samples were eluted on a hydrophilic interaction LC column (ZIC-pHILIC, Merck) and analysed on a Thermo Q-Exactive (Thermo Scientific), operated in polarity switching mode to record both positive and negative ionization mode data for each sample. A pooled sample, prepared by combining an aliquot from each individual sample, was injected every 5th injection to confirm the stability of the analysis. The raw MS data were processed in a non-targeted way, using a pipeline consisting of XCMS (peak picking) and MZMatch (grouping and filtering).^26–30^

### 2.5 Statistics

Categorical variables are presented as absolute numbers and percentages and compared by χ^2^ test. Quantitative variables were tested for normal distribution in the whole cohort and in individual groups by graphical plot and Kolmogorov–Smirnov test; they are presented as mean±SD, or median and inter-quartile range in case of a skewed distribution. Parametric and nonparametric statistics were used for normally and non-normally distributed variables, respectively. In particular, one-way analysis of variance (ANOVA) or Kruskal–Wallis test was used to compare anthropometric, clinical, and biochemical data across study groups, with Tukey or Dunn’s as *post hoc* tests, as appropriate; crude correlations were ascertained by Pearson or Spearman tests.

Multivariable-adjusted comparisons (ANCOVA) and linear regression models included significant covariates identified at comparison of Na-intake groups, that is, age, sex, BMI, aldosterone, and systolic BP, upon appropriate transformation to attain normal distribution. Little’s missing completely at random (MCAR) test was used beforehand to test the assumption that variables were missing completely at random, including the above covariates and urinary (u-) Na^+^ excretion in the analysis; no imputation methods were adopted and missing data were excluded, with valid numbers for each analysis reported in the manuscript.

Slopes of the regression lines for FEs, assessing tubular Na^+^ and water handling, were compared between high and low Na^+^ intake groups using the extra-sum-of-squares *F* test, with automatic outliers exclusion (conservative Q for ROUT approach set at 0.5%) and normality of residuals confirmed with Kolmogorov–Smirnov test.

For metabolomics, the intensity of peaks with a matching database formula^31^ was log-transformed and *t*-test comparisons were conducted between high and low Na^+^ intake groups, using a moderated linear model. The *P*-values for the targeted and non-targeted analysis were corrected to control the false discovery rate.^32^

The α level was set at 0.05 and all statistical tests were 2-tailed. SPSS (version 25, IBM) and Prism (version 8.02, GraphPad Software) were used for the analysis.

## 3. Results

### 3.1 Cohort characteristics

Out of 1464 patients, we excluded 592 in whom washout of confounding medications was not possible and 106 who received a final diagnosis of secondary hypertension (Supplementary material online, *Figure S1*). The final study cohort therefore included data from 766 patients, almost exclusively of Caucasian ethnicity. Their clinical and biochemical general characteristics by Na^+^ intake groups are reported in *Table 1*. Of note, pre-defined 24 u-Na^+^ cut-off values for group allocation closely approximated the extreme quintiles of the distribution (102 and 219 mmol/day for 20th and 80th percentile, respectively).

Patients on high Na^+^ intake were generally younger, had a higher BMI and similar BP values, although more frequently required doxazosin on top of a first-line calcium channel blocker, compared with other Na^+^ groups. Prevalence of diabetes and/or CKD (KDIGO stage ≥ 3) in the cohort was low (<4% for both) and did not differ across study groups.

While plasma Na^+^ and K^+^ did not differ, plasma aldosterone was higher with low Na^+^ intake (*P* = 0.040). Renin showed a similar trend, which reached statistical significance upon correction for age and sex, significant predictors at multivariate regression analysis (Supplementary material online, *Tables S2* and *S3* and *Figures S5* and *S6*). Overall, the ARR did not differ across study groups. In the 24 h urine, higher Na^+^ excretion was paralleled by a higher K^+^ excretion and total urinary volume (*P* < 0.001 for both).

**Table 2:** Renal function by Na^+^ intake group

	*n*_valid_	Whole cohort	Low-Na^+^	Medium-Na^+^	High-Na^+^	*P*	*p*_adj_
p-Creatinine (μmol/L)	664	73 (63–84)	69 (59–78)	73 (63–84)*	75 (68–85)*	0.002	0.027
u-Creatinine (mmol/L)	325	7.0 (5.0–10.4)	5.7 (4.1–9.4)	6.7 (4.6–10.3)	9.1 (6.3–10.5)*,†	0.001	<0.001
24h u-Creatinine excretion (mmol/day)	325	12.0 (8.8–15.6)	8.8 (7.1–11.9)	11.7 (8.9–15.3)*	16.0 (13.9–19.7)*,†	<0.001	<0.001
Estimated GFR:							
Creatinine clearance (mL/min)	303	119.2 (93.0–151.1)	100.5 (75.1–117.8)	116.6 (91.7–142.4)*	150.3 (125.6–178.1)*,†	<0.001	<0.001
Creatinine clearance/BSA (mL/min/1.73 m^2^)	249	108.6 (86.7–128.8)	94.1 (69.9–118.8)	103.8 (86.9–126.0)*	127.5 (108.3–147.8)*,†	<0.001	<0.001
Glomerular hyperfiltration—*n*/tot (%)		48/249 (19.3)	5/61 (8.2)	21/128 (16.4)	22/60 (36.7)*,†	<0.001	
eGFR—CKD-EPI (mL/min/1.73 m^2^)	664	98.4 (86.6–107.6)	98.8 (86.8–106.0)	97.0 (85.4–106.3)	100.8 (91.4–111.7)*,†	0.001	0.02
Glomerular hyperfiltration—*n*/tot (%)		8/664 (1.2)	0/122 (0)	3/405 (0.7)	5/137 (3.6)*	0.011	
p-Urea (mmol/L)	498	4.8 (4.0–5.7)	4.7 (4.0–5.6)	4.7 (3.9–5.6)	5.1 (4.2–6.1)†	0.026	0.724
u-Urea (mmol/L)	173	196.6 (127.2–271.3)	145.5 (100.3–237.4)	177.4 (125.9–257.8)	260.2 (177.9–314.1)*,†	0.001	0.015
24h-u-Urea excretion (mmol/day)	173	337.5 (239.6–499.0)	242.9 (178.3–343.0)	332.1 (244.8–453.9)*	496.8 (373.0–610.6)*,†	<0.001	<0.001

p-, plasma; u-, urine; GFR, glomerular filtration rate; BSA, body surface area; *p*_adj_, analysis of variance adjusted for age, sex, systolic blood pressure, BMI, and aldosterone.

* *Post hoc* tests: *P* < 0.05 versus low-Na^+^; †*P* < 0.05 versus medium-Na^+^.

### 3.2 Renal Na^+^ and water handling

**Figure 1:**
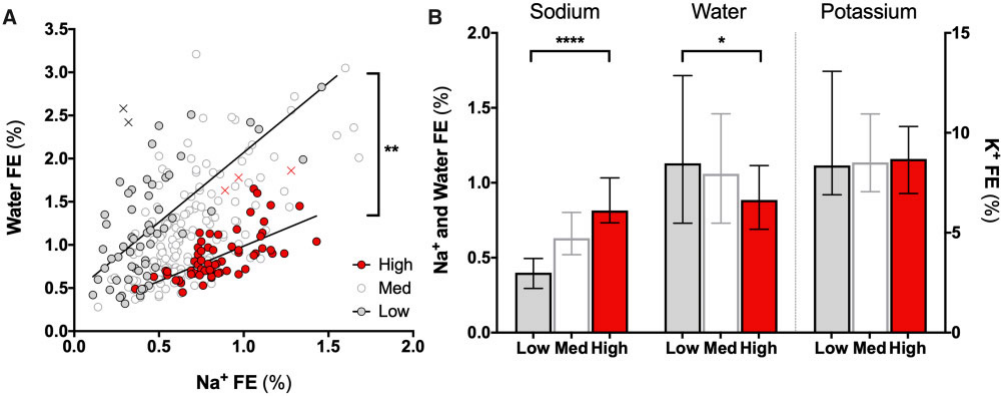
Renal Na and water handling upon differential Na^+^ intake. (*A*) The positive association between Water and Na^+^ fractional excretions (FE) reflects he osmotic effect of Na^+^, driving a parallel excretion of accompanying water (*n*_tot_ = 282); however, the slope of this association is steeper at low (white dots; *n* = 61) compared to high (red dots; *n* = 63) Na^+^ intake [1.62 (95% CI: 1.14–2.09) vs. 0.81 (95% CI: 0.56–1.06), respectively; *P* = 0.005, extra-sum-of-squares *F* test; X indicate automatically excluded outliers (ROUT approach, Q = 0.5%) from high and low Na^+^ intake groups]. (*B*) With increasing Na^+^ intake, Na^+^ FE increases while water FE decreases (Kruskall–Wallis test: *P* < 0.001 and *P* = 0.017, respectively; Dunn’s *post hoc* test results on top of bars); no significant difference across groups was noted for K^+^. Cases are the same as for panel *A*; *n* > 60 per bar. Data are shown as median and IQR; *P < 0.05, **P < 0.01; ****P < 0.0001.

We first tested the association between the FEs of Na^+^ and water (*n* = 282, Little’s MCAR test Sig. = 0.185 and *n* = 303, Sig. = 0.163, respectively), as expression of the traditional osmotic natriuresis mechanism challenged by Rakova *et al.*^10^ In the context of an expected positive correlation (Spearman ρ = 0.402, *P* < 0.001), the slope of the regression line was steeper at low compared to high salt intake (*P* = 0.005; *Figure 1A*). In fact, the FE of Na^+^ increased with increasing Na^+^ intake [*P* < 0.001 across groups; *post hoc* low vs. high: 0.39% (0.30–0.47) vs. 0.81% (0.73–0.98), *P* < 0.001] while that of water decreased [*P* = 0.016 across groups; *post hoc* low vs. high: 1.13% (0.73–1.72) vs. 0.89% (0.69–1.12), *P* = 0.015; *Figure 1B*]. No such difference was observed for K^+^ FE (*P* = 0.892).

To dissect the determinants of the above differences, we compared GFRs and absolute measures of tubular activity across study groups.

### 3.3 Excess Na excretion is paralleled by glomerular hyperfiltration

In keeping with evidence of a higher excretion of creatinine in the 24 h, BSA-corrected (and uncorrected) creatinine clearance was greater in high than in medium- and low-Na^+^ intake (*P* < 0.001 across groups; *n* = 249; Little’s MCAR test Sig. = 0.221; *Table 2*). In the high Na^+^ group 36.7% of the patients met the definition of ‘glomerular hyperfiltration’ according to a commonly used cut-off (135 mL/min/1.73 m^2^),^33^^,^^34^ compared to 16.4% and 8.2% in the medium and low Na^+^ intake group, respectively (χ^2^, *P* < 0.001). Similar trends were confirmed with use of CKD-EPI formula, although with lower estimates of eGFR and of glomerular hyperfiltration prevalence, accordingly (*Table 2* and Supplementary material online, *Table S4*).

An independent association between 24 h urinary Na^+^ excretion and creatinine clearance was confirmed at multivariate regression analysis, after correction for age, sex, systolic BP, BMI, and aldosterone (*P* < 0.001). In a regression model including also 24 h urinary K^+^, as an independent surrogate marker for global food intake based on the above demonstration of a constant tubule handling, both variables remained significant predictors (*P* = 0.003 and *P* < 0.001, respectively; Supplementary material online, *Tables S5* and *S6*).

### 3.4 High Na^+^ intake increases tubular reabsorption and renal energy expenditure

**Figure 2:**
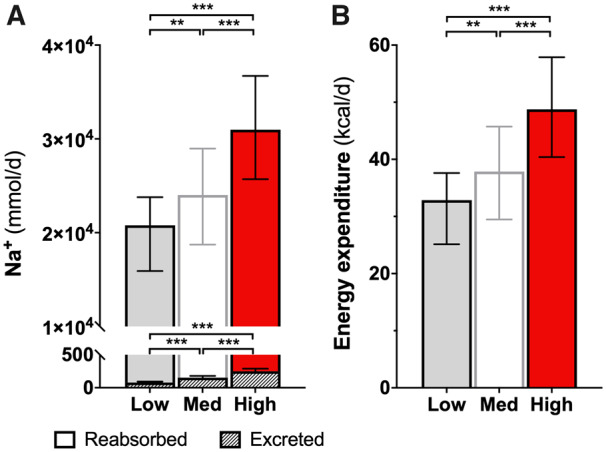
Absolute Na^+^ excretion and reabsorption and energy cost. (*A*) The total excreted Na^+^ (shaded bars) is a trivial proportion of the reabsorbed amount; the latter is much higher upon high Na^+^ intake and resulted in 18 kcal of estimated excess energy expenditure per day (*B*). Data are shown as median and IQR; *n*_tot_ = 282, *n*_low Na_ = 61, *n*_high Na_ = 63. Kruskall–Wallis and Dunn’s *post hoc* tests: **P < 0.01; ***P < 0.001.

Although the FE was higher with high Na^+^ intake, the total amount of Na^+^ filtered by the glomerulus and reabsorbed by the tubules per day was far larger compared to medium and low Na^+^ intake (*P* < 0.001, regardless of adjustment for the above covariates, including stratification by sex; *Figure 2A* and Supplementary material online, *Table S4*).

The estimated energy cost for this excess reabsorptive tubular activity is shown in *Figure 2B* [Δ high vs. low Na^+^ intake groups = 18 (12–24) kcal/day; *P* < 0.001].

Urinary norepinephrine excretion, as a surrogate measure of renal sympathetic nerve activity contribution to tubular Na^+^ reabsorption,^35^^,^^36^ increased across groups of Na^+^ intake (*P* < 0.001) and was independently associated with 24 h-u-Na^+^ excretion (Supplementary material online, *Figure S7* and Tables S7–S10).

### 3.5 Re-setting of nitrogen balance and metabolic signatures

While 24 h-urine excretion data suggested a higher daily loss of both creatinine and urea with high Na^+^ intake (*P* ≤ 0.001 for both), plasma values increased or did not differ, respectively (*Table 2*). No difference in the FE of urea was observed across groups (*P* = 0.724). Overall, this suggested a global re-setting of the nitrogen balance.

**Figure 3:**
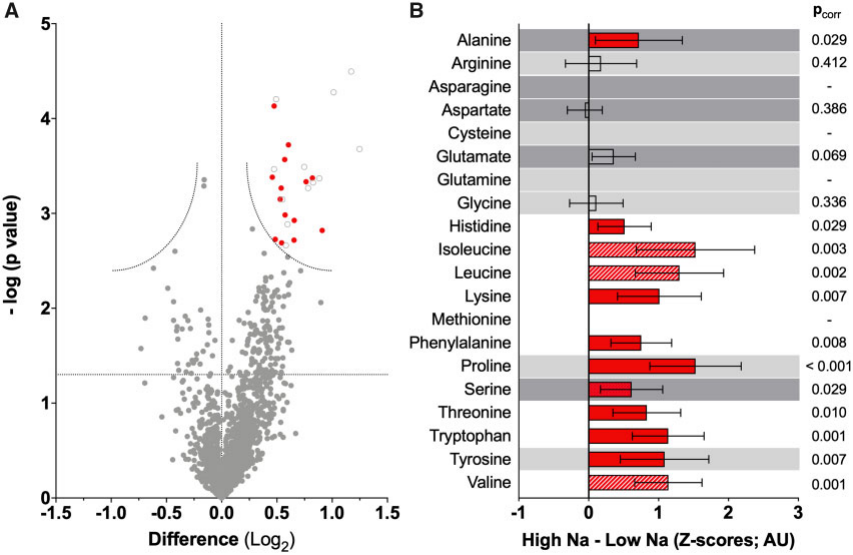
Metabolomics signature. (*A*) Volcano plot showing the metabolomics comparison between high and low (reference) Na^+^ intake (*n*_tot_ = 67 subjects; *n*_lowNa_ = 32, *n*_HighNa_ = 35). *Y*-axis: uncorrected *P*-values; curved lines: multiple comparisons-corrected significance (*p*_corr_<0.05). Red dots: intermediates/end products of the urea cycle or protein catabolism; empty dots: other identified metabolites (Supplementary material online, Table S11). (*B*) Comparative profile of plasma amino acids LC/MS signals (mean, 95% CI); for visualization, the x scale was made homogeneous by *Z*-score transformation, based on mean and SD from the low Na^+^ group, as a reference; *n* = 35 (high Na). Background: white = essential amino acids; light grey = conditionally essential, dark grey = dispensable amino acids. Bars: dashed for branched-chain amino acids; red for significance (*p*_corr_ < 0.05). Peaks for asparagine, cysteine, glutamine, and methionine could not be unequivocally identified. For all statistical comparisons, log-transformed peak intensities of the *n* = 67 subjects were compared by *t*-tests with a false discovery rate correction.

Analysis of non-targeted metabolomics showed that the majority of metabolites significantly increased in the high Na^+^ (*n* = 35) compared to low Na^+^ group (*n* = 32) entailed intermediates or end products of protein catabolism (i.e. dipeptides, single amino acids, or their derivatives) or urea cycle (*N*-acetyl-l-glutamate 5-semialdehyde: fold change = 1.52, *p*_corr_ = 0.02; *Figure 3B*). In particular, plasma levels of all detectable amino acids (including the branched-chain leucine, isoleucine, and valine and with the exception of few conditionally essential or non-essential amino acids) were increased with high Na^+^ intake (*Figure 3B*).

Other significantly increased metabolites identified by the non-targeted approach included products of triglycerides and fatty acid metabolism (diacylglycerols and acyl-glycines), acyl-carnitines, and some industrial food/tobacco-related compounds (*Figure 3A*). A complete list of significantly different metabolites is provided in the see Supplementary material online, *Table S11*.

While no obvious shift in carbohydrate metabolism was observed at metabolomics analysis, higher Na^+^ intake was associated with higher plasma glucose [*n* = 171; high Na^+^: 4.9 (4.6–5.4) mmol/L, medium Na^+^: 4.7 (4.3–5.0) mmol/L, low Na^+^: 4.5 (4.2–5.0) mmol/L; *P* = 0.004] and insulin [*n* = 47; high Na^+^: 7.46 (4.75–11.80) µU/mL, medium Na^+^: 5.81 (3.79–9.24) µU/mL, low Na^+^: 4.89 (3.72–8.04) µU/mL; *P* = 0.001]; however, these associations did not persist at multivariable analysis, where BMI stood out as the strongest common independent predictor (*r* = 0.38, *P* = 0.002 and *r* = 0.48, *P* < 0.001, respectively; Supplementary material online, *Tables S12–S15*).

### 3.6 Excess cortisol excretion upon high Na^+^ intake

We additionally explored whether cortisol excess could be a primary drive for muscle catabolism upon high Na^+^ intake, as suggested previously.^11^ In our study, UFC increased across groups of Na^+^ intake [*n* = 137, Little’s MCAR test Sig. = 0.161; low Na^+^: 63 (36–72) nmol, medium Na^+^: 60 (47–86) nmol, high Na^+^: 86 (75–139) nmol; *P* < 0.001]. The positive association of UFC with 24 h-u-Na^+^ excretion persisted at multivariable analysis after correction for age, sex, systolic BP, BMI, and aldosterone (*P* = 0.017), and also for creatinine clearance (*P* = 0.035); however, the latter stood out as the strongest independent predictor (*P* < 0.001; Supplementary material online, *Tables S16* and *S17*).

Morning plasma-cortisol showed an opposite trend [*n* = 658; low Na^+^: 246 (203–309) nmol/L, medium Na^+^: 238 (194–294) nmol/L, high Na^+^: 217 (171–294) nmol/L; *P* = 0.047]; no independent association with 24 h-u-Na^+^ was observed (*P* = 0.284; Supplementary material online, *Tables S18* and *S19*).

## 4. Discussion

Our study examined the hypothesis that high Na^+^ intake could induce mechanisms of water preservation and adversely affect metabolic signatures, surrogates for global cardiovascular risk, in a large real-life population of hypertensive patients. This idea was first proposed by Titze *et al.*^10^^,^^11^ based on the diet-controlled experimental settings of a rodent study and a long-term simulated space flight of 10 healthy subjects. Our results, obtained under normal dietary conditions and salt excretions comparable to those reported in other populations,^17^ showed that the higher excretion of Na^+^ was indeed coupled with a higher excretion of water, in keeping with the classic concept of osmotic natriuresis. However, we identified opposite trends relative to their filtered amount: while the FE increased for Na^+^ upon high Na^+^ intake, it decreased for water and this was paralleled by a plasma metabolomic signature consistent with protein catabolism and with the results obtained by Titze *et al.* in rodent models. These findings indicate that kidneys can effectively dissociate Na^+^ and water handling upon high Na^+^ intake and that the associated catabolic state, likely participating in this water preservation mechanism, could independently affect the risk of cardiovascular disease.

Previous clinical studies suggested a different body water handling, assessed as body weight change and diuretic response upon salt load and depletion, between salt-sensitive and salt-resistant (insensitive) subjects.^37^ Our study, albeit lacking a formal assessment of salt sensitivity, rather focused on the renal-specific differential regulation of Na^+^/water excretion and its correlates, independently of BP (Supplementary material online, *Tables S5* and *S6*). Such fine regulation relies upon a larger amount of processed urine and a higher GFR. A recent meta-analysis identified this association also in interventional trials,^38^ but was limited in its conclusions by the heterogeneity in study designs, populations, and approaches to estimation of GFR. In the present study, we employed a standardized screening approach and a rigorous protocol that included washout of confounding medications, the use of 24 h urine collections for the assessment of both Na^+^ and creatinine excretion and non-targeted metabolomics. In a large sample size, we showed that a considerable proportion of patients exhibit glomerular hyperfiltration upon high Na^+^ intake: although estimates differ depending on the method used for GFR assessment,^24^ this proportion is consistently higher compared to both medium and low Na^+^ groups. Glomerular hyperfiltration, traditionally linked to obesity and diabetes,^33^ is a recognized marker of early kidney damage, precedes microalbuminuria and/or decline in renal function and predicts cardiovascular events.^34^ Our study suggests Na^+^ intake, independent of BP values and of a surrogate for total food consumption, as a key determinant in the pathogenesis of glomerular hyperfiltration in hypertension, thus confirming previous suggestions from a smaller study.^39^

While higher filtration carries the ultimate advantage of more precise distal regulation of solutes,^40^ not only has it negative long-term prognostic implications but also comes at the cost of a much higher tubular activity at more proximal segments (*Figure 2*). The increase in Na^+^ reabsorption in response to the increased filtered Na^+^ load is known as glomerulo-tubular balance and is primarily active via Na^+^/K^+^ ATPase, with ancillary passive mechanisms facilitated by changes in tubular, interstitial, and capillary physical forces. The extra Na^+^/K^+^ ATPase activity implicates a higher oxygen and energy consumption.^41^ Pruijm *et al*.^42^ found that one week of high-Na^+^ diet reduced renal medullary oxygenation in both normotensive and hypertensive subjects by using blood oxygen level-dependent (BOLD) MRI, thus pointing to a higher oxygen extraction by tubular cells, which ultimately supports our contentions.

Our estimate of the excess energy cost upon high versus low Na^+^ intake was 18 kcal. This is a rough (potentially over- or under-) estimate, based on a stoichiometry value that averages different tubular segments with different activity and was validated in ‘standard’ conditions.^25^ Albeit imprecise, it offers an order of magnitude that corresponds approximately to 4.5 g of protein or 2 g of fat per day. These values should be considered in a lifespan or population perspective. Of note, with the due haemo- and tubule-dynamic corrections required between species, the magnitude of this energy cost well justifies the weight loss observed in high Na^+^-fed mice when their total caloric food intake was paired with low Na^+^-fed controls.^11^ In that animal model, the catabolic state primarily oriented towards protein degradation and muscle loss served to generate both endogenous water and osmotically active urea. Overall, these mechanisms allowed water preservation against Na^+^ excess and a potentially volume-depleting osmotic diuresis.^11^ In our study, a similar re-setting in nitrogen balance was observed: along with evidence of massive excretions of urea and creatinine upon high Na^+^ intake, we identified a catabolic signature at non-targeted metabolomics, mostly entailing intermediates or end products of the urea cycle or protein catabolism. In particular, plasma levels of all the identifiable essential amino acids were increased, thus ruling out endogenous generation or the sole renal recycling as sources for their excess. To the best our knowledge, this is the first metabolomics approach to the topic of salt balance in humans.

Based on the increase in 24 h urinary glucocorticoid excretion upon high Na^+^ diet, Titze *et al.* suggested a Na^+^-induced subclinical hypercortisolism as the intermediate determinant of the above catabolic state.^10^^,^^11^ In our human cohort, we could not confirm a cortisol increase in plasma. The UFC excretion, although increased and independently associated with Na^+^ excretion, had GFR as its strongest predictor and its increase was clinically trivial, particularly when GFR-adjusted. This appears in keeping with human physiological studies assessing the renal clearance of plasma cortisol,^43^ or UFC excretion in other glomerular hyperfiltration-associated conditions, like obesity^44^ or simple water load.^45^ In fact, only subtle increases in adrenal cortisol secretion in response to Na^+^ loading, possibly due to the cross stimulation of the hypothalamic–pituitary–adrenal axis by the water-preserving vasopressin,^46^ were observed by Ehrlich *et al.*^47^ despite marked changes in urinary excretion. As discussed above, we propose that glomerular hyperfiltration and the consequent glomerulo-tubular balance *per se* would suffice to induce extra energy requirements and the development of a catabolic state.

The main limitation of our study is its cross-sectional nature, unsuitable to prove causality. However, mechanistic evidence of a metabolic shift had already been provided in preclinical models^11^ and our study has the advantage to offer a perspective on real-life, where caloric and/or water intake is not restricted or fixed. Obviously, this prevented discrimination as to how this energy cost, predominantly but not exclusively in the form of protein, was paid. Although the association between Na^+^ intake and renal haemodynamics/energetics was independent of K^+^ excretion, a crude surrogate for total food intake, this could reflect catabolism of either endogenous (muscle mass) or exogenous (dietary excess) sources. Based on the higher BMI and a larger urea clearance in our high Na^+^ group, we speculate that both options could be exploited to different degrees in different individuals, according to multiple determinants. These would include cultural and socio-economical aspects, favouring or limiting food access. In this regard, the *per-protocol* controlled caloric intake in the 10 healthy male cosmonauts could account for the reduced excretion and increased recycling of urea observed in the original study by Rakova *et al.*,^10^ but not in our real-life cohort. Similarly, it could account for the discrepancy of those preclinical data with the recent findings by Juraschek *et al*.,^48^ who found increased thirst upon high Na^+^ diet in a secondary analysis of the randomized DASH-Sodium trial, where caloric intake was not fixed. Juraschek *et al*. also provided reassurance against a putative weight gain effect of low salt intake, if endogenous sources were the only available for Na^+^-induced catabolism. At variance, they could not draw definite conclusions regarding high Na^+^ intake, since the trial failed at adjusting the energy intake of participants to maintain a stable weight, further confounded by unmeasured fluid retention.

Overall, the importance of an altered caloric balance is such that the excess exploitation of exogenous protein sources would ultimately result in excess fat deposition regardless of food relative composition.^49^ Therefore, excess food consumption and lean mass loss, alternative or complementary sources for the catabolic state observed in our study, are both likely to favour a long-term adverse insulin-resistant metabolic phenotype, universally associated to worse cardiovascular outcomes (please see Graphical Abstract).

Additional limitations of the study include: use of a single 24 h urine collection, which might not accurately estimate an individual’s usual long-term daily sodium intake; missing data that potentially reduced the power of some analyses, for example, for UFC; the lack of a formal BP ‘salt-sensitivity’ assessment in the protocol, as mentioned, as well as the ethnicity distribution of our almost exclusively Caucasian cohort, which might require validation in other groups like African–Americans. However, estimates of intake by 24 h urine collections in properly sized groups are not significantly affected by random variability across individuals,^17^ particularly when they are not instructed to artificially change their dietary habits. Moreover, use of the same 24 urine samples for the assessment of all renal handling parameters provides an intra-patient control, with all fluctuations going in the same biological direction and, overall, levelling off in large numbers. Finally, absence of evidence of systematic bias and strong statistical significance for most of our results makes type II error or any considerable impact of missing data on the overall message unlikely.

In conclusion, our results confirmed the activation of water-preserving mechanisms upon high Na^+^ intake in a large real-life cohort of patients with essential hypertension. These mechanisms appear to involve glomerular hyperfiltration, enhanced glomerulo-tubular balance, increased tubular energy expenditure, and protein catabolism, with broad implications on cardiovascular risk. The preferential endogenous or exogenous source of protein to compensate for these energy costs in different subjects/populations remains to be established in interventional studies, where caloric intake is controlled but not restricted. However, it is already tempting to speculate that specific dietary strategies and/or novel medications like SGLT-2 inhibitors, reducing both glomerular hyperfiltration and the energy-demanding tubular Na^+^ reabsorption, could favourably impact the metabolic consequences of excess Na^+^ intake and the global risk profile of hypertensive patients. 

**Summary (graphical abstract):** High salt intake is traditionally linked to cardiovascular risk via its effect on blood pressure (BP, in grey). Preclinical studies recently described a metabolic shift toward catabolism upon high sodium (Na^+^) diet, ultimately favouring body water preservation and possibly impacting cardiovascular risk, irrespective of BP. In a large cohort of hypertensive patients we confirmed that kidneys preserve water and excrete sodium excess upon high salt intake; this was associated with glomerular hyperfiltration, higher tubular workload and a plasma metabolomic signature suggestive of protein catabolism. Muscle loss and/or excess food consumption, paralleled by adverse renal haemodynamics in a putative vicious circle, could represent a novel BP-independent link between salt intake and cardiovascular disease.

## Data availability

Access to fully deidentified data generated or analysed during this study can be provided upon reasonable request from qualified researchers trained in human subject confidentiality protocols.

**Translational perspective**

We herein show that high Na intake can adversely impact not only blood pressure control but also renal function and metabolic balance in hypertensive patients. At variance with experimental preclinical studies, the catabolism of proteins appears to include also exogenous sources. Interventional studies where caloric intake is controlled but not restricted may identify preferential metabolic handling in different subjects/populations and test the effect of specific dietary strategies. Similarly, the potential benefit of medications like sodium–glucose cotransporter (SGLT)-2 inhibitors, which are known to reduce both glomerular hyperfiltration and excess tubular Na^+^ reabsorption, in non-diabetic hypertensive patients deserves further investigation in relation to the described Na^+^-reno-metabolic mechanisms.

## Supplementary material

Supplementary material is available at *Cardiovascular Research* online.

## Authors’ contributions

G.R. had full access to all of the data in the study and take responsibility for the integrity of the data and the accuracy of the data analysis. Concept and design: G.R. and C.D.; recruitment of participants and diagnosis: G.R., G.M., C.B., V.B., M.C., T.M.S., and G.P.R.; acquisition, analysis, or interpretation of biochemical data: G.R., G.M., S.L., G.C., G.A., C.B., V.B., M.C., T.M.S., L.L., A.P., P.W., M.P., G.P.R., and C.D.; acquisition, analysis, or interpretation of metabolomic data: G.R., G.C., G.B., S.M., R.D., and C.D.; drafting of the manuscript: G.R.; critical revision of the manuscript for important intellectual content: G.R., G.M., G.B., S.M., A.M., R.M.T., M.C.P., G.P.R., and C.D.; statistical analysis: G.R., S.M., R.D., and C.D.; supervision: R.M.T., M.C.P., C.D., and G.P.R.; obtained funding: G.R., G.B., R.M.T., C.D., and G.P.R.

## Supplementary Material

cvaa205_Supplementary_Data
