# Supplementary material for: High sodium intake, glomerular hyperfiltration, and protein catabolism in patients with essential hypertension
Source: Cardiovasc Res. 2020 Jul 16;117(5):1372–81. doi: 10.1093/cvr/cvaa205 (PMC8064429; doi:10.1093/cvr/cvaa205)
Supplement: cvaa205_Supplementary_Data [file cvaa205_supplementary_data.docx]

**ONLINE SUPPLEMENT**

**High Sodium Intake, Glomerular Hyperfiltration and Protein Catabolism**

**in Patients with Essential Hypertension**

*Rossitto Giacomo, MD et al.*

**EXPANDED METHODS**

**Patients and diagnostic protocol**

**Laboratory methods**

**Renal function and energetics**

**Metabolomics**

**Statistics**

**Figure S1. Study flowchart**

**EXPANDED DATASET**

**Figure S2. Plasma Creatinine - Passing and Bablok regression**

**Figure S3. Plasma Urea - Passing and Bablok regression**

**Figure S4. Urinary Creatinine - Passing and Bablok regression**

**Table S1. Creatinine and Urea Data by Na+ Intake Group - Dataset comparison.**

**RENIN DISTRIBUTION – covariates and adjustment**

**Table S2. Plasma renin activity (PRA; 2012-2015)**

**Figure S5. Plasma renin activity (PRA; 2012-2015)**

**Table S3. Direct renin concentration (DRC; 2015-2017)**

**Figure S6. Direct renin concentration (DRC; 2015-2017)**

**IMPACT OF NA INTAKE ON RENAL FUNCTION, BY SEX**

**Table S4. Renal function by Na+ intake group, stratified by sex**

**CREATININE CLEARANCE**

**Table S5. Creatinine Clearance and Sodium Excretion – Correlations**

**Table S6. Creatinine Clearance and Sodium Excretion – Multivariable regression**

**CATECHOLAMINE / METANEPHRINE EXCRETION**

**Figure S7. 24h-urinary Catecholamine / Metanephrine Excretion**

**Table S7. Norepinephrine (NE) and Sodium Excretion – Correlations**

**Table S8. Norepinephrine (NE) and Sodium Excretion – Multivariable regression**

**Table S9. Normetanephrine (NM) and Sodium Excretion – Correlations**

**Table S10. Normetanephrine (NM) and Sodium Excretion – Multivariable regression**

**METABOLOMICS**

**Table S11. Different Peaks (High Na^+^ vs Low Na^+^)**

**GLUCIDIC METABOLISM**

**Table S12. Glucose and Sodium Excretion – Correlations**

**Table S13. Insulin and Sodium Excretion – Correlations**

**Table S14. Glucose and Sodium Excretion – Multivariable regression**

**Table S15. Insulin and Sodium Excretion – Multivariable regression**

**CORTISOL**

**Table S16. Urinary Free Cortisol and Sodium Excretion – Correlations**

**Table S17. Urinary Free Cortisol and Sodium Excretion – Multivariable regression**

**Table S18. Morning Plasma Cortisol and Sodium Excretion – Correlations**

**Table S19. Morning Plasma Cortisol and Sodium Excretion – Multivariable regression**

**ONLINE SUPPLEMENT REFERENCES and GRAPHIC ABSTRACT CREDITS**

**STROBE STATEMENT – CHECKLIST**

# EXPANDED METHODS

## Patients and diagnostic protocol

Clinical and biochemical data from consecutive consenting patients referred to the tertiary Hypertension Center of the University of Padua between 2012 and December 2017 were reviewed. Patients provided written informed consent for data and samples use as part of a local biobank for diagnosis of adrenal disease (Prot.1925P/2009), later implemented for the ongoing multi-centre ENSAT-HT study (<http://www.ensat-ht.eu/>; local ethics approval protocol 3998/AO/16), to which both institutions (University of Padua, University of Glasgow) participate.

Our study included cases undergoing a biochemical screening for secondary causes of hypertension, entailing assessment of plasma electrolytes, aldosterone, renin and cortisol and 24h urinary Na^+^ and K^+^ excretion; additional biochemical data, including renal function, albuminuria, 24h creatinine excretion, 24h urinary catecholamines/metanephrines and urinary free cortisol were measured as indicated by guidelines^1-4^ and/or at the discretion of the requesting physician. Patients were not instructed to change their usual dietary habits before the screening. They were asked to complete a 24h urine collection, reporting start and stop times: collections were used for both clinical and study purposes when included ≥ 22 hours and a volume ≥ 500 ml, with no significant loss during collection reported. On the morning of completion, blood sampling was performed after one-hour in semi-supine position, between 8 and 10 AM. A sample from the 24h urine container and an additional blood sample in EDTA were collected upon availability of the technician in charge on the day; the EDTA sample was centrifuged at room temperature for separation of plasma and frozen with the urine for long term storage in the local biobank. If requested, an additional sample from the 24h urine container was acidified with HCl and used for the measurement of urinary norepinephrine, epinephrine, normetanephrine, and metanephrine excretion. Attended office blood pressure values were determined at the time of the screening from 2 or 3 consecutive, brachial blood pressure readings with an automated calibrated sphygmomanometer, as per guidelines.^1^ Diabetes status was defined by self-reported diagnosis by a clinician or use of a diabetes medication; chronic kidney disease was defined by an estimated glomerular filtration rate <60 mL/min/1.73 m2 with CKD-EPI formula.^5^

The biochemical screening was systematically performed while off antihypertensive treatment or, if patients were already treated, after appropriate washout from confounding agents and switch to calcium channels blockers and/or doxazosin, as per guidelines;^2^ in patients on a mineralocorticoid receptor antagonist (spironolactone, canrenone or potassium canreonate), or on agents affecting the renin-angiotensin-aldosterone system (diuretics, beta-blockers, angiotensin-converting enzyme inhibitors and angiotensin II type 1 receptor antagonists) at least six-weeks or two-weeks wash-out period was required, respectively. To minimise the confounding effect of medications, cases who could not tolerate this change or for whom it was judged unsafe/impractical by the physician in charge were excluded from this analysis. Similarly, cases with reported/biochemical evidence of intramuscular or intravenous steroid use or abuse and cases with a final diagnosis of secondary hypertension after appropriate work-up (biochemistry, anatomical/functional imaging, adrenal/renal vein sampling) and follow-up at the time of data-lock (1^st^ January 2019) were excluded. Upon database scrutiny, diagnoses of essential hypertension were confirmed with physicians in charge (GR, GM, VB, MC, TMS, and GPR).

Patients with a conclusive diagnosis of essential hypertension were grouped according to classes of Na^+^ intake. Intake estimates were based on 24u-excretion, a ‘gold standard’ approach only minimally affected by within-individual day-to-day variability when a sufficient number of participants is included,^6^ and commonly used cut-offs: low ≤2.3g/d (100 mmol/d); medium 2.3-5g/d; high >5g/d (216 mmol/d).^7,8^

## Laboratory methods

Plasma and urinary electrolytes, plasma renin, aldosterone and cortisol, as well as additional routine biochemistry as appropriate and detailed above, were all measured at the time of the secondary hypertension screening in an International Standard Organisation (ISO) 15189 accredited clinical laboratory (University of Padua).

Plasma and urinary electrolytes were measured using ion‑selective electrodes (ISE indirect Na-K-Cl Cobas, Roche Diagnostics GmbH, Mannheim). The measuring range for plasma were: Na^+^ 80‑180 mmol/L, K^+^ 1.5‑10.0 mmol/L, Cl^-^ 60‑140 mmol/L. For urine samples, the measuring range were: Na^+^ 20‑250 mmol/L, K^+^ 3‑100 mmol/L, Cl^-^ 20‑250 mmol/L. The intermediate imprecision for Na^+^ was less than 2% and 5% for plasma and urine, respectively. The intermediate imprecision for K^+^ was less than 3% for both matrices.

Renin was measured as plasma renin activity (PRA) until April 2015 and direct renin concentration thereafter (DRC). PRA was measured using a competitive radioimmunoassay kit (RIA, Beckman Coulter kit ) based on in vitro generation of Ang I per hour, as reported,^9^ with intra-assay and inter-assay coefficient of variation within 8% and 10%. DRC was measured by chemiluminescence (LIAISON^®^ Direct Renin kit, DiaSorin, Saluggia, Italy), with intra- and inter-assay coefficient of variations of 2.4% and 8.4%, respectively, in the range of 4 - 282 mIU·L^-1^; the normal range of DRC values in a multi-ethnic cohort of healthy normotensive subjects was 2.8-39.9 mIU·L^-1^ (5^th^-95^th^ percentile) in the supine position (DiaSorin, Instruction for use). Renin data for the two cohorts are reported separately in the manuscript and/or the supplemental material. Plasma aldosterone concentration (PAC) was measured by a chemiluminescence competitive immunoassay (LIAISON^®^, Aldosterone kit, DiaSorin, Saluggia, Italy) the limit of quantification was 1,91 ng/dL and the analytical measurement range up to 100 ng/dL, as stated by manufacturer. The declared intra-assay and inter-assay coefficients of variation were less than 5% and 11%, respectively. Aldosterone-to-renin ratio (ARR) criteria for primary aldosteronism and further diagnostic work-up were ≥ 40 ng/dl/ng/ml/h with PRA or ≥ 4 ng/dl/mUI/l with DRC, as per guidelines and previously reported.^2, 9, 10^

Plasma cortisol concentration (PCC) was measured by a chemiluminescence competitive immunoassay (Immulite 2000 cortisol, Siemens Healthcare Diagnostics Products Ltd., Gwynedd, UK). The declared analytical sensitivity was 5,5 nmol/l, with the analytical range up to 1380 nmol/L. The stated intra-assay and inter-assay coefficients of variation were less than 10%. Urinary 24h free cortisol (UFC) was measured by a home-made LC-MS/MS, validated according to ISO15189:2012. The analytical measurement range was 5-6325 nmol/L, with a lower limit of quantification of 5 nmol/L. Imprecision was less than 10% for both intra- and inter- assay coefficients of variation.^11^ Cases with cortisol values below the lower quantification limits (5,5 nmol/l for PCC and 5 nmol/l for UFC) or with 24h UFC > 197 nmol/d, which suggest exogenous steroid use/abuse and diagnostic for Cushing syndrome with 100% specificity and sensitivity,^12^ respectively, were excluded from the analysis.

Urinary catecholamines were measured by HPLC with electrochemical detection with a CE-IVD kit (Chromsystems^®^, Chromsystems Instruments & Chemicals GmbH, Gräfelfing, Germany). The declared limits of quantification were 2.5, 1.6 and 3.6 µg/L for adrenaline, noradrenaline and dopamine respectively. Imprecision was less than 5% for both intra- and inter-assay coefficients of variation, for all the analytes. Urinary metanephrines were determined by HPLC with electrochemical detection with a CE-IVD kit (Chromsystems^®^, Chromsystems Instruments & Chemicals GmbH, Gräfelfing, Germany). The declared limits of quantification were 5 µg/L for normetanephrine, 10 µg/L for metanephrine and 11 µg/L for 3-methoxytyramine, with linear range up to at least 1000 µg/L for normetanephrine and at least 5000 µg/L for metanephrine and 3-methoxytyramine. Intra- and inter-assays imprecisions were less than 5% for all the analytes.

Plasma and 24h urinary urea and creatinine were not routinely determined in all screened patients; the available biochemical dataset was expanded by analysing urine samples stored in the local biobank at the time of the screening. In particular: a) urea in plasma and urine was measured by a kinetic test with urease and glutamate dehydrogenase (UREAL Cobas, Roche Diagnostics GmbH, Mannheim), with a measuring range of 0.5‑40 mmol/L and1‑2000 mmol/L for plasma and urine, respectively, and imprecision < 2% and < 3% for plasma and urine, respectively; b) creatinine in plasma and urine was measured by an enzymatic method (CREP Cobas, Roche Diagnostics GmbH, Mannheim), with a measuring range of 5‑2700 μmol/L and 0.1‑54 mmol/L for plasma and urine, respectively, and total imprecision < 5% for both plasma and urine samples. Results available from the time of the screening served for comparison and validation of the entire *de novo* measured batches, according to the Passing & Bablok method.^13^ Briefly, this is a linear regression procedure with no special assumptions regarding the distribution of the samples, estimating systematic, proportional and random differences and controlling for linear model validity (Cusum test). Results for plasma creatinine and urea and for urinary creatinine for the validation of the plasma and urine batches, respectively, are reported in this Supplement (Figure S1-3; Table S1); no urinary values for urea were available from the time of the screening. Estimated regression equations were used for correction of results.

## Renal function and energetics

Urine samples collected over the 24h immediately before plasma standardised sampling were used for estimation of glomerular and tubular function, according to standard equations. In particular, glomerular filtration rate (GFR) was estimated by creatinine clearance:

$$C_{Cr}= \frac{uCreatinine \times24h urinary Volume}{pCreatinine \times24 \times60 min}$$

Body surface area (BSA)-corrected creatinine clearance was calculated as:

$$C_{Cr-BSA corrected}= \frac{C_{Cr} \times1.73}{\sqrt{(Height \times{Weight)}/{3600}}}$$

GFR was also estimated according to CKD-EPI formula (eGFR),^5^ for comparison with values routinely used in clinical practice.

Tubular handling of Na^+^, K^+^ and water were assessed by their respective fractional excretions (FE), i.e. the ratio between the excreted and the filtered amount. In particular, for Na^+^ and K^+^, they were calculated as:

$${FE}_{Na}=\frac{Na excreted}{Na filtered}= \frac{\left( pCreatinine \times uNa \right)}{\left( pNa \times uCreatinine \right)}$$

$${FE}_{K}=\frac{K excreted}{K filtered}= \frac{\left( pCreatinine \times uK \right)}{\left( pK \times uCreatinine \right)}$$

later multiplied by 100 to express them as percentages; *p* and *u* prefixes indicate plasma and urinary concentrations, respectively.

Fractional excretion of water is volume of water that appears as urine compared to the filtered amount, thus:

$${FE}_{Water}= \frac{urine flow rate (V)}{GFR} =\frac{V}{\frac{uCreatinine x V}{pCreatinine}}= \frac{pCreatinine}{uCreatinine}$$

similarly multiplied by 100 for data presentation as percentages.

GFR estimation by creatinine clearance (ml/min) and FE_Na_ allowed calculation of the absolute amount (mmol/d) of filtered [GFR x pNa x 24h x 60 min], reabsorbed [1 – (filtered x FE_Na_)] and excreted Na^+^ [filtered x FE_Na_]. Based on known stoichiometry of renal transepithelial Na^+^ transport to ATP (4.6 Na^+^/ATP) and assuming 7.3 kcal/mol for the free energy equivalent of ATP,^14^ we calculated estimated energy expenditure for the tubular reabsorptive activity [(reabsorbed Na^+^/d x 7.3 kcal/mol ATP)/4.6 Na^+^/ATP; kcal/d].

## Metabolomics

A sub-cohort of patients from the low and the high Na^+^ intake groups and available EDTA-plasma samples stored in Padua biobank from the time of screening were compared for plasma metabolomics signatures. Samples were extracted with HPLC grade chloroform/methanol/water (1:3:1 v/v; Merck, Sigma-Aldrich). A pooled sample was prepared by combining an aliquot from each individual sample into a single sample and they were then stored at −80 °C until analysis by liquid chromatography – mass spectrometry (LC-MS). Samples were eluted on a hydrophilic interaction liquid chromatography (HILIC) column (ZIC-pHILIC, 150 x 4.6mm, Merck) using a linear gradient (A = 20mM ammonium carbonate in water, B = acetonitrile) over 26 minutes at 300µL/min as follows: time 0 min = 20% A, 80% B; 15 min = 80% A, 20% B; 15 min = 95% A, 5% B; 17 min = 95% A, 5% B; 17 min = 20% A, 80% B; 26 min = 20% A, 80% B. The column was maintained at 40^o^C.

For MS analysis a Thermo Q-Exactive (Thermo Scientific, Hemel Hampstead) was operated in polarity switching mode to record both positive and negative ionisation mode data for each sample. Full scan data was recorded across the m/z range 70-1000. Prior to running any samples, the instrument was calibrated according to the manufacturers specifications and QC’d using in house controls to ensure the system was stable. Before experimental sample analysis, 3 mixes of authentic standards were analysed. These standards were used to confirm the identity of metabolites where applicable based upon accurate mass and retention time. All masses matched are accurate to 3ppm or less. 6 “burn in” pooled samples were run at the beginning of the experimental sample analysis to condition the column and ensure that it was stable upon commencement of the analysis. The pooled sample was injected every 5^th^ injection and these samples were used to confirm the stability of the instrument over the course of the analysis. After the experimental sample analysis, fragmentation data was collected in both positive and negative ionisation mode for the pooled sample. This data was again used to confirm the identities of metabolites where applicable. The raw mass spectrometry data was converted to mzXML via msconvert from ProteoWizard^15^ and processed in an untargeted way, using a pipeline consisting of XCMS^16^ (peak picking) and MZMatch^17^ (grouping and filtering) through PiMP.^18^ Signals of interest were identified within the output statistics were calculated to determine differences between the groups as described below.

## Statistics

Categorical variables are presented with absolute numbers and percentages and compared by χ2 test. Quantitative variables were tested for normal distribution in the whole cohort and in individual groups by graphical plot and Kolmogorov–Smirnov test; they are presented as mean ± SD, or median and interquartile range in case of a skewed distribution. Parametric and nonparametric statistics were used for normally and non-normally distributed variables, respectively. In particular, one-way analysis of variance or Kruskall-Wallis test was used to compare anthropometric, clinical and biochemical data across study groups, with Tukey or Dunn’s as post-hoc tests, as appropriate; crude correlations were ascertained by Pearson or Spearman tests, as detailed in the manuscript and Supplement. Multivariable-adjusted comparisons (ANCOVA) and multiple linear regression models included significant covariates identified at comparison of Na-intake groups, i.e. age, sex, BMI, PAC, systolic blood pressure, and additional covariates when appropriate for each specific analysis as detailed in the manuscript/Supplement; covariates were appropriately transformed to attain normal distribution. Little’s MCAR test was used beforehand to test the assumption that variables were missing completely at random according to the above covariates and u-Na^+^ excretion; no imputation methods were adopted and missing data were excluded, with valid numbers for each analysis reported in the manuscript.

Slopes of the regression lines for fractional excretions, assessing tubular Na^+^ and water handling, were compared between high and low Na^+^ intake groups using the extra-sum-of-squares F test, with automatic outliers exclusion (conservative Q for ROUT approach set at 0.5%)^19^ and normality of residuals confirmed with Kolmogorov–Smirnov test.

For metabolomics, only peaks that matched a known formula from KEGG,^20^ HMDB^21^ or Lipidmaps^22^ were carried through for later analysis. Peak intensities were log transformed (using base 2). Limma^23^ was used to perform moderated t-tests between high and low Na^+^ intake groups for each of the peaks. For the non-targeted analysis, p-values were corrected to q-values.^24^ Due to the likelihood of correlated responses, for the targeted amino-acid sub-analysis, a two stage false-discovery-rate correction was used.^25^

The α level was set at 0.05 and all statistical tests were 2-tailed. SPSS (version 25, IBM) and Prism (version 8.02, GraphPad Software) were used for the analysis.

**Figure S1. STUDY FLOWCHART**

Flowchart identifying the final study cohort, the distribution into classes of Na^+^ intake according to 24h-uNa^+^ excretion and the subcohort of patients for non-targeted metabolomics comparison.

EH = essential hypertension; FENA and FEwater = fractional excretion of Na and water, respectively; eGFR = estimated glomerular filtration rate according to CKD-EPI equation; 24h-UFC = 24h urinary free cortisol excretion.

# EXPANDED DATASET – samples quality check

**Figure S2. PLASMA CREATININE - Passing and Bablok regression**

| Sample size | 498 |
| --- | --- |

|  | Variable X | Variable Y |
| --- | --- | --- |
| Lowest value | 46.0000 | 44.0000 |
| Highest value | 187.0000 | 200.0000 |
| Arithmetic mean | 74.0100 | 75.7811 |
| Median | 71.0000 | 74.0000 |
| Standard deviation | 18.2431 | 18.0300 |
| Standard error of the mean | 0.8175 | 0.8079 |

**Regression Equation**

| y = 2.000000  +  1.000000  x | | |
| --- | --- | --- |
| **Systematic differences** | | |
| Intercept A (95% CI) | | 2.0000 (-1.2381 to 6.2222) |
| **Proportional differences** | | |
| Slope B (95% CI) | | 1.0000 (0.9444 to 1.0476) |
|  | |  |
| **Random differences** | | |
| Residual Standard Deviation (RSD) ± 1.96 RSD Interval | | 8.5926 (-16.8415 to 16.8415) |
|  | |  |
| **Linear model validity** | | |
| Cusum test for linearity | No significant deviation from linearity (P=0.74) | |

**Spearman rank correlation coefficient**

| Correlation coefficient | 0.722 |
| --- | --- |
| Significance level | P<0.0001 |
| 95% CI | 0.677 to 0.761 |

Left, scatter diagram and regression line; Y axis = plasma creatinine measured at the time of screening; X axis = plasma creatinine measured on samples stored at the time of screening; red solid line = regression line with confidence interval (red dashed lines), black dotted line = identity line (x=y). Right, residual plot for goodness of fit, showing a linear relationship with no artificial pattern; red dots = outliers (Passing-Bablok regression is a non-parametric procedure and not influenced by the presence of one or relative few outliers).

**Figure S3. PLASMA UREA - Passing and Bablok regression**

| Sample size | 152 |
| --- | --- |

|  | Variable X | Variable Y |
| --- | --- | --- |
| Lowest value | 2.9000 | 2.4000 |
| Highest value | 12.8000 | 11.8000 |
| Arithmetic mean | 5.5243 | 5.2546 |
| Median | 5.3500 | 5.0000 |
| Standard deviation | 1.5137 | 1.4718 |
| Standard error of the mean | 0.1228 | 0.1194 |

**Regression Equation**

| y = -0.400000  +  1.000000  x | | |
| --- | --- | --- |
| **Systematic differences** | | |
| Intercept A (95% CI) | | -0.4000 (-0.5237 to 0.02857) |
|  | |  |
| **Proportional differences** | | |
| Slope B (95% CI) | | 1.0000 (0.9286 to 1.0263) |
| **Random differences** | | |
| Residual Standard Deviation (RSD) ± 1.96 RSD Interval | | 0.4917 (-0.9637 to 0.9637) |
|  | |  |
| **Linear model validity** | | |
| Cusum test for linearity | No significant deviation from linearity (P=0.63) | |

**Spearman rank correlation coefficient**

| Correlation coefficient | 0.842 |
| --- | --- |
| Significance level | P<0.0001 |
| 95% CI | 0.789 to 0.883 |

Left, scatter diagram and regression line; X axis = plasma urea measured at the time of screening; Y axis = plasma urea measured on samples stored at the time of screening; red solid line = regression line with confidence interval (red dashed lines), black dotted line = identity line (x=y). Right, residual plot for goodness of fit, showing a linear relationship with no artificial pattern; red dots = outliers (Passing-Bablok regression is a non-parametric procedure and not influenced by the presence of one or relative few outliers)

**Figure S4. URINARY CREATININE - Passing and Bablok regression**

| Sample size | 57 |
| --- | --- |

|  | Variable X | Variable Y |
| --- | --- | --- |
| Lowest value | 0.7560 | 2.3000 |
| Highest value | 17.6100 | 20.7000 |
| Arithmetic mean | 7.2149 | 8.0614 |
| Median | 6.2790 | 7.0000 |
| Standard deviation | 3.9760 | 4.0895 |
| Standard error of the mean | 0.5266 | 0.5417 |

**Regression Equation**

| y = 0.537369  +  1.021494  x | | | |
| --- | --- | --- | --- |
| **Systematic differences** | | | |
| Intercept A (95% CI) | | 0.5374 (0.08877 to 0.8494) | |
| **Proportional differences** | | | |
| Slope B (95% CI) | | 1.0215 (0.9703 to 1.0837) | |
| **Random differences** | | | |
| Residual Standard Deviation (RSD) ± 1.96 RSD Interval | | | 0.8306 (-1.6280 to 1.6280) |
|  | | |  |
| **Linear model validity** | | | |
| Cusum test for linearity | No significant deviation from linearity (P=0.93) | | |

**Spearman rank correlation coefficient**

| Correlation coefficient | 0.962 |
| --- | --- |
| Significance level | P<0.0001 |
| 95% CI | 0.936 to 0.977 |

Left, scatter diagram and regression line; Y axis = 24h urinary creatinine measured at the time of screening; X axis = 24h urinary creatinine measured on samples stored at the time of screening; red solid line = regression line with confidence interval (red dashed lines), black dotted line = identity line (x=y). Right, residual plot for goodness of fit, showing a linear relationship with no artificial pattern; red dots = outliers (Passing-Bablok regression is a non-parametric procedure and not influenced by the presence of one or relative few outliers)

|  |  | **n** | **whole cohort** | **Low-Na^+^** | **Middle-Na^+^** | **High-Na^+^** | **p** |
| --- | --- | --- | --- | --- | --- | --- | --- |
| **original** | **p-Creatinine (μmol/l)** | 494 | 73 [63-84] | 70 [59-78] | 72 [63-85] | 76 [68-85] | 0.022 |
| **final** | **p-Creatinine (μmol/l)** | 664 | 73 [63-84] | 69 [59-78] | 73 [63-84] | 75 [68-85] | 0.002 |
| **original** | **eGFR – CKD-EPI (ml/min/1.73m^2^)** | 494 | 97.6 [86.5-107.0] | 97.7 [84.6-106.1] | 96.5 [85.4-106.3] | 102.2 [91.7-111.8] | 0.003 |
| **final** | **eGFR – CKD-EPI (ml/min/1.73m^2^)** | 664 | 98.4 [86.6-107.6] | 98.8 [86.8-106.0] | 97.0 [85.4-106.3] | 100.8 [91.4-111.7] | 0.001 |
| **original** | **p-Urea (mmol/l)** | 151 | 4.8 [4.1-5.7] | 4.7 [4.0-5.2] | 4.8 [3.9-5.7] | 5.0 [4.2-6.1] | 0.274 |
| **final** | **p-Urea (mmol/l)** | 498 | 4.8 [4.0-5.7] | 4.7 [4.0-5.6] | 4.7 [3.9-5.6] | 5.1 [4.2-6.1] | 0.026 |
| **original** | **u-Creatinine (mmol/l)** | 195 | 7.7 [5.2-10.9] | 6.9 [4.1-11.4] | 7.4 [5.1-10.9] | 9.9 [7.5-10.9] | 0.039 |
| **final** | **u-Creatinine (mmol/l)** | 325 | 7.0 [5.0-10.4] | 5.7 [4.1-9.4] | 6.7 [4.6-10.3] | 9.1 [6.3-10.5] | 0.001 |
| **original** | **u-Urea (mmol/l)** | n/a | - | - | - | - | - |
| **final** | **u-Urea (mmol/l)** | 173 | 196.6 [127.2-271.3] | 145.5 [100.3-237.4] | 177.4 [125.9-257.8] | 260.2 [177.9-314.1] | 0.001 |

**Table S1. CREATININE AND UREA DATA BY NA^+^ INTAKE GROUP - Original and expanded dataset comparison.**

Original = biochemical dataset measured at the time of screening (Padua); final = expanded dataset after inclusion of samples stored at the time of screening but measured later, upon Passing and Bablok validation (see above). p- = plasma; u- = urine. eGFR - CKD-EPI = Glomerular filtration rate estimated with CKD-EPI equation. p across groups, α = 0.05, two-tailed.

# RENIN DISTRIBUTION – covariates and adjustment

## Tables S2. Plasma renin activity (PRA; 2012-2015)

| Correlations | | **PRA** | **SEX** | **uNa24h** | **Age** | **BMI** | **SBP** |
| --- | --- | --- | --- | --- | --- | --- | --- |
| Pearson Correlation | **PRA** (Log) | 1.000 | -.109 | .012 | -.224 | .026 | -.098 |
|  | **SEX**: 1M, 2F | -.109 | 1.000 | -.357 | .099 | -.264 | .035 |
|  | **uNa24h** (Square root) | .012 | -.357 | 1.000 | -.081 | .310 | -.089 |
|  | **Age** | -.224 | .099 | -.081 | 1.000 | .116 | .312 |
|  | **BMI** (Log) | .026 | -.264 | .310 | .116 | 1.000 | .022 |
|  | **SBP** | -.098 | .035 | -.089 | .312 | .022 | 1.000 |
| Sig. (1-tailed) | **PRA** (Log) |  | .027 | .414 | .000 | .378 | .071 |
|  | **SEX**: 1M, 2F | .027 |  | .000 | .003 | .000 | .189 |
|  | **uNa24h** (Square root) | .414 | .000 |  | .012 | .000 | .012 |
|  | **Age** | .000 | .003 | .012 |  | .004 | .000 |
|  | **BMI** (Log) | .378 | .000 | .000 | .004 |  | .314 |
|  | **SBP** | .071 | .189 | .012 | .000 | .314 |  |
| N | **PRA** (Log) | 313 | 313 | 313 | 313 | 144 | 225 |
|  | **SEX**: 1M, 2F | 313 | 766 | 766 | 766 | 537 | 647 |
|  | **uNa24h** (Square root) | 313 | 766 | 766 | 766 | 537 | 647 |
|  | **Age** | 313 | 766 | 766 | 766 | 537 | 647 |
|  | **BMI** (Log) | 144 | 537 | 537 | 537 | 537 | 483 |
|  | **SBP** | 225 | 647 | 647 | 647 | 483 | 647 |

| **Model**  Dependent variable: **PRA** | | **Unstandardized Coefficients** | | **Standardized Coefficients** | **t** | **Sig.** | **95% CI for B** | | **Collinearity Statistics** | |
| --- | --- | --- | --- | --- | --- | --- | --- | --- | --- | --- |
|  |  | **B** | **Std. Error** | **Beta** |  |  | **Lower Bound** | **Upper Bound** | **Tolerance** | **VIF** |
| R^2^=.062 | (Constant) | .104 | .798 |  | .130 | .897 | -1.474 | 1.682 |  |  |
|  | **SEX**: | -.082 | .078 | -.095 | -1.053 | .294 | -.237 | .072 | .837 | 1.195 |
|  | **uNa24h** | -.009 | .015 | -.056 | -.608 | .544 | -.038 | .020 | .812 | 1.232 |
|  | **Age** | -.007 | .003 | -.214 | -2.425 | .017 | -.013 | -.001 | .872 | 1.146 |
|  | **BMI** | .258 | .524 | .044 | .493 | .623 | -.778 | 1.295 | .853 | 1.173 |
|  | **SBP** | -.001 | .002 | -.034 | -.390 | .697 | -.005 | .003 | .898 | 1.114 |
| R^2^=.058 | (Constant) | .230 | .159 |  | 1.448 | .150 | -.084 | .543 |  |  |
|  | **SEX** | -.076 | .071 | -.088 | -1.067 | .288 | -.217 | .065 | .990 | 1.010 |
|  | **Age** | -.007 | .003 | -.216 | -2.626 | .010 | -.012 | -.002 | .990 | 1.010 |

| **ANCOVA - Tests of Between-Subjects Effects** | | | | | | |
| --- | --- | --- | --- | --- | --- | --- |
| Dependent Variable: | PRA (Log) |  |  |  |  |  |
| **Source** | **Type III Sum of Squares** | **df** | **Mean Square** | **F** | **Sig.** | **Partial Eta Squared** |
| **Corrected Model** | 4.144^a^ | 4 | 1.036 | 5.947 | .000 | .072 |
| **Intercept** | 1.126 | 1 | 1.126 | 6.463 | .012 | .021 |
| **Age** | 2.867 | 1 | 2.867 | 16.456 | .000 | .051 |
| **Sex** | .612 | 1 | .612 | 3.511 | .062 | .011 |
| **Na Intake (L-M-H)** | .908 | 2 | .454 | 2.606 | .075 | .017 |
| **Error** | 53.656 | 308 | .174 |  |  |  |
| **Total** | 71.854 | 313 |  |  |  |  |
| **Corrected Total** | 57.800 | 312 |  |  |  |  |
| a. R Squared = .072 (Adjusted R Squared = .060) | | | | | | |

## Figure S5. Plasma renin activity (PRA; 2012-2015)

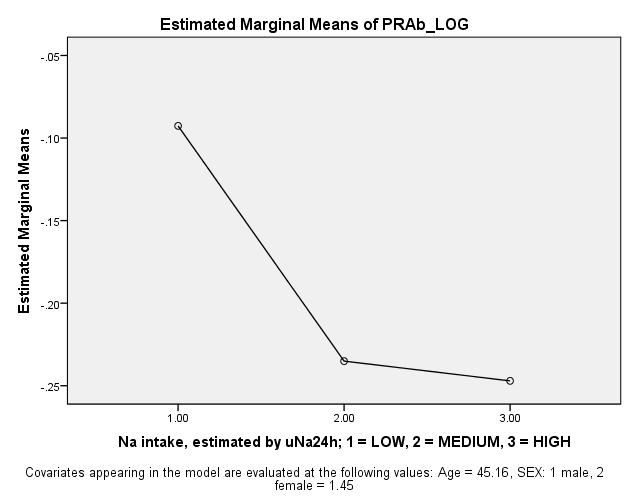


Plasma renin activity (PRA) distribution across groups of Na^+^ intake. Top: left, violin plot; right, dot plot; please note cases of low-renin hypertension, spread across all groups (area highlighted in red). Bottom: age- and sex- corrected PRA estimates across groups (ANCOVA p = 0.075).

## Tables S3. Direct renin concentration (DRC; 2015-2017)

| Correlations | | **DRC** | **SEX** | **uNa24h** | **Age** | **BMI** | **SBP** |
| --- | --- | --- | --- | --- | --- | --- | --- |
| Pearson Correlation | **DRC** (Log) | 1.000 | -.202 | -.064 | -.173 | .090 | -.101 |
|  | **SEX**: 1M, 2F | -.202 | 1.000 | -.357 | .099 | -.264 | .035 |
|  | **uNa24h** (Square root) | -.064 | -.357 | 1.000 | -.081 | .310 | -.089 |
|  | **Age** | -.173 | .099 | -.081 | 1.000 | .116 | .312 |
|  | **BMI** (Log) | .090 | -.264 | .310 | .116 | 1.000 | .022 |
|  | **SBP** | -.101 | .035 | -.089 | .312 | .022 | 1.000 |
| Sig. (1-tailed) | **DRC** (Log) |  | .000 | .087 | .000 | .037 | .019 |
|  | **SEX**: 1M, 2F | .000 |  | .000 | .003 | .000 | .189 |
|  | **uNa24h** (Square root) | .087 | .000 |  | .012 | .000 | .012 |
|  | **Age** | .000 | .003 | .012 |  | .004 | .000 |
|  | **BMI** (Log) | .037 | .000 | .000 | .004 |  | .314 |
|  | **SBP** | .019 | .189 | .012 | .000 | .314 |  |
| N | **DRC** (Log) | 452 | 452 | 452 | 452 | 392 | 421 |
|  | **SEX**: 1M, 2F | 452 | 766 | 766 | 766 | 537 | 647 |
|  | **uNa24h** (Square root) | 452 | 766 | 766 | 766 | 537 | 647 |
|  | **Age** | 452 | 766 | 766 | 766 | 537 | 647 |
|  | **BMI** (Log) | 392 | 537 | 537 | 537 | 537 | 483 |
|  | **SBP** | 421 | 647 | 647 | 647 | 483 | 647 |

| **Model**  Dependent variable: **DRC** | | **Unstandardized Coefficients** | | **Standardized Coefficients** | **t** | **Sig.** | **95% CI for B** | | **Collinearity Statistics** | |
| --- | --- | --- | --- | --- | --- | --- | --- | --- | --- | --- |
|  |  | **B** | **Std. Error** | **Beta** |  |  | **Lower Bound** | **Upper Bound** | **Tolerance** | **VIF** |
| R^2^=.062 | (Constant) | 1.066 | .453 |  | 2.356 | .019 | .176 | 1.956 |  |  |
|  | **SEX**: | -.189 | .044 | -.225 | -4.275 | .000 | -.276 | -.102 | .837 | 1.195 |
|  | **uNa24h** | -.031 | .008 | -.198 | -3.702 | .000 | -.047 | -.014 | .812 | 1.232 |
|  | **Age** | -.005 | .002 | -.160 | -3.104 | .002 | -.008 | -.002 | .872 | 1.146 |
|  | **BMI** | .640 | .297 | .112 | 2.152 | .032 | .055 | 1.224 | .853 | 1.173 |
|  | **SBP** | -.001 | .001 | -.063 | -1.238 | .217 | -.004 | .001 | .898 | 1.114 |
| R^2^=.058 | (Constant) | 1.313 | .086 |  | 15.294 | .000 | 1.145 | 1.482 |  |  |
|  | **SEX** | -.157 | .039 | -.187 | -4.076 | .000 | -.233 | -.081 | .990 | 1.010 |
|  | **Age** | -.005 | .001 | -.155 | -3.369 | .001 | -.008 | -.002 | .990 | 1.010 |

| **ANCOVA - Tests of Between-Subjects Effects** | | | | | | |
| --- | --- | --- | --- | --- | --- | --- |
| Dependent Variable: | PRAb (Log) |  |  |  |  |  |
| **Source** | **Type III Sum of Squares** | **df** | **Mean Square** | **F** | **Sig.** | **Partial Eta Squared** |
| **Corrected Model** | 6.420^a^ | 4 | 1.605 | 9.955 | .000 | .082 |
| **Intercept** | 37.305 | 1 | 37.305 | 231.376 | .000 | .341 |
| **Age** | 1.741 | 1 | 1.741 | 10.800 | .001 | .024 |
| **Sex** | 3.596 | 1 | 3.596 | 22.304 | .000 | .048 |
| **Na Intake (L-M-H)** | 1.172 | 2 | .586 | 3.635 | .027 | .016 |
| **Error** | 72.071 | 447 | .161 |  |  |  |
| **Total** | 409.968 | 452 |  |  |  |  |
| **Corrected Total** | 78.491 | 451 |  |  |  |  |
| a. R Squared = .072 (Adjusted R Squared = .060) | | | | | | |

## Figure S6. Direct renin concentration (DRC; 2015-2017)

**
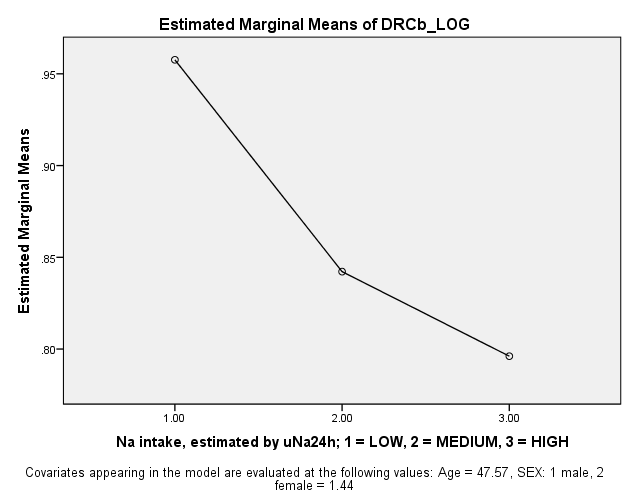
**

Direct renin concentration (DRC) distribution across groups of Na^+^ intake. Top: left, violin plot; right, dot plot; please note cases of low-renin hypertension, spread across all groups (area highlighted in red). Bottom: age- and sex- corrected DRC estimates across groups (ANCOVA p = 0.027).

**IMPACT OF NA INTAKE ON RENAL FUNCTION, BY SEX**

## Table S4. Renal function by Na^+^ intake group, stratified by sex

|  | **n_valid_** | **Low-Na^+^** | **Medium-Na^+^** | **High-Na^+^** | **p** | **p_adj_** |
| --- | --- | --- | --- | --- | --- | --- |
| **Males** | | | | | | |
| **p-Creatinine** (μmol/l) | 376 | 81 [74-93] | 82 [73-92] | 78 [71-86] | 0.016 | 0.073 |
| **eGFR – CKD-EPI** (ml/min/1.73m^2^) | 376 | 97.8 [88.7-104.6] | 96.7 [84.7-106.4] | 100.8 [90.5-111.8] ^†^ | 0.008 | 0.012 |
| **24h u-Creatinine excretion** (mmol/d) | 177 | 10.9 [7.7-13.9] | 15.2 [12.3-17.9] * | 16.3 [14.0-19.8] *^†^ | < 0.001 | < 0.001 |
| **Creatinine Clearance** (ml/min) | 166 | 105.7 [72.6-127.2] | 131.2 [104.2-156.7] * | 154.7 [125.6-178.1] *^†^ | < 0.001 | < 0.001 |
| **Creatinine Clearance/BSA** (ml/min/1.73m^2^) | 132 | 89.0 [64.4-115.1] | 103.5 [87.6-120.5] | 127.4 [108.4-147.2] *^†^ | < 0.001 | < 0.001 |
| **FE Na^+^** (%) | 154 | 0.40 [0.32-0.57] | 0.61 [0.50-0.81] * | 0.81 [0.73-1.00] *^†^ | < 0.001 | 0.002 |
| **Energy for Na^+^** **reabsorption** (kcal/d) | 154 | 33.5 [21.9-38.6] | 42.5 [33.3-52.0] * | 49.2 [40.3-57.9] *^†^ | < 0.001 | 0.001 |
| **Females** | | | | | | |
| **p-Creatinine** (μmol/l) | 288 | 62 [56-70] | 63 [58-69] | 63 [56-68] | 0.537 | 0.649 |
| **eGFR – CKD-EPI** (ml/min/1.73m^2^) | 288 | 99.2 [86.2-106.4] | 97.1 [85.8-106.2] | 102.8 [94.7-112.8] | 0.232 | 0.753 |
| **24h u-Creatinine excretion** (mmol/d) | 148 | 8.4 [7.0-10.1] | 9.8 [8.0-11.3] * | 12.9 [10.5-15.1] *^†^ | < 0.001 | 0.007 |
| **Creatinine Clearance** (ml/min) | 137 | 97.9 [73.8-116.1] | 103.7 [85.5-128.1] | 131.7 [122.6-221.7] *^†^ | 0.002 | < 0.001 |
| **Creatinine Clearance/BSA** (ml/min/1.73m^2^) | 117 | 102.3 [71.7-119.5] | 103.8 [84.7-126.9] | 129.5 [103.9-209.4] * | 0.054 | 0.038 |
| **FE Na^+^** (%) | 128 | 0.40 [0.26-0.49] | 0.67 [0.55-0.80] * | 0.85 [0.60-1.14] * | < 0.001 | 0.004 |
| **Energy for Na^+^** **reabsorption** (kcal/d) | 128 | 32.4 [25.6-37.7] | 33.5 [27.3-41.3] | 41.8 [38.4-70.7] *^†^ | 0.007 | 0.036 |

p- = plasma; u- = urine. GFR = Glomerular filtration rate. BSA = body surface area. p_adj_ = analysis of variance adjusted for age, sex, systolic blood pressure, BMI and aldosterone. Post-hoc tests:* p < 0.05 vs Low-Na^+^; ^†^ p < 0.05 vs Medium-Na^+^ . p_adj_ = sig. corrected for age, sex, systolic blood pressure, BMI, and aldosterone.

**CREATININE CLEARANCE AND SODIUM EXCRETION**

## Table S5. Creatinine Clearance and Sodium Excretion - Correlations

| **Correlations** | | **Cr Cl** | **Age** | **SEX 1M, 2F** | **SBP** | **BMI** | **PAC** | **uNa24h** | **uK24h** |
| --- | --- | --- | --- | --- | --- | --- | --- | --- | --- |
| Pearson Correlation | **Cr Clearance** | 1.000 | -.109 | -.203 | -.087 | .203 | .040 | .358 | .346 |
|  | **Age** | -.109 | 1.000 | .099 | .312 | .116 | -.010 | -.081 | .136 |
|  | **SEX: 1M, 2F** | -.203 | .099 | 1.000 | .035 | -.264 | -.098 | -.357 | -.279 |
|  | **SBP** | -.087 | .312 | .035 | 1.000 | .022 | .016 | -.089 | .019 |
|  | **BMI** (Log) | .203 | .116 | -.264 | .022 | 1.000 | .077 | .310 | .196 |
|  | **PAC** (Log) | .040 | -.010 | -.098 | .016 | .077 | 1.000 | -.060 | .063 |
|  | **uNa24h** (Square root) | .358 | -.081 | -.357 | -.089 | .310 | -.060 | 1.000 | .402 |
|  | **uK24h** (Square root) | .346 | .136 | -.279 | .019 | .196 | .063 | .402 | 1.000 |
| Sig. (1-tailed) | **Cr Clearance** |  | .029 | .000 | .079 | .001 | .244 | .000 | .000 |
|  | **Age** | .029 |  | .003 | .000 | .004 | .388 | .012 | .000 |
|  | **SEX: 1M, 2F** | .000 | .003 |  | .189 | .000 | .003 | .000 | .000 |
|  | **SBP** | .079 | .000 | .189 |  | .314 | .343 | .012 | .321 |
|  | **BMI** (Log) | .001 | .004 | .000 | .314 |  | .038 | .000 | .000 |
|  | **PAC** (Log) | .244 | .388 | .003 | .343 | .038 |  | .049 | .046 |
|  | **uNa24h** (Square root) | .000 | .012 | .000 | .012 | .000 | .049 |  | .000 |
|  | **uK24h** (Square root) | .000 | .000 | .000 | .321 | .000 | .046 | .000 |  |
| N | **Cr Clearance** | 303 | 303 | 303 | 269 | 249 | 303 | 303 | 297 |
|  | **Age** | 303 | 766 | 766 | 647 | 537 | 766 | 766 | 723 |
|  | **SEX: 1M, 2F** | 303 | 766 | 766 | 647 | 537 | 766 | 766 | 723 |
|  | **SBP** | 269 | 647 | 647 | 647 | 483 | 647 | 647 | 609 |
|  | **BMI** (Log) | 249 | 537 | 537 | 483 | 537 | 537 | 537 | 516 |
|  | **PAC** (Log) | 303 | 766 | 766 | 647 | 537 | 766 | 766 | 723 |
|  | **uNa24h** (Square root) | 303 | 766 | 766 | 647 | 537 | 766 | 766 | 723 |
|  | **uK24h** (Square root) | 297 | 723 | 723 | 609 | 516 | 723 | 723 | 723 |

Cr Clearance = Creatinine Clearance (ml/min); SEX:1M,2F = 1 male, 2 female; SBP = systolic blood pressure; BMI = Body mass index (Kg/m^2^); PAC = plasma aldosterone concentration (pmol/l); uNa24h = 24h urinary Na^+^ excretion (mmol/d); uK24h = 24h urinary K^+^ excretion (mmol/d).

## Table S6. Creatinine Clearance and Sodium Excretion – Multivariable regression

| **Model**  Dependent variable: **Cr Clearance** | | **Unstandardized Coefficients** | | **Standardized Coefficients** | **t** | **Sig.** | **95.0% Confidence Interval for B** | | **Collinearity Statistics** | |
| --- | --- | --- | --- | --- | --- | --- | --- | --- | --- | --- |
|  |  | **B** | **Std. Error** | **Beta** |  |  | **Lower Bound** | **Upper Bound** | **Tolerance** | **VIF** |
| R^2^ = .15 | (Constant) | -36.097 | 76.509 |  | -.472 | .637 | -186.805 | 114.611 |  |  |
|  | **Age** | -.314 | .252 | -.079 | -1.246 | .214 | -.809 | .182 | .872 | 1.147 |
|  | **SEX: 1M, 2F** | -5.746 | 6.773 | -.055 | -.848 | .397 | -19.088 | 7.596 | .827 | 1.209 |
|  | **SBP** | -.105 | .180 | -.036 | -.581 | .562 | -.459 | .250 | .898 | 1.114 |
|  | **BMI** (Log) | 72.335 | 45.377 | .102 | 1.594 | .112 | -17.050 | 161.720 | .847 | 1.181 |
|  | **PAC** (Log) | 11.042 | 14.944 | .044 | .739 | .461 | -18.395 | 40.479 | .973 | 1.028 |
|  | **uNa24h** (Square root) | 5.715 | 1.263 | .299 | 4.524 | .000 | 3.227 | 8.204 | .801 | 1.249 |
| R2 = .20 | (Constant) | -72.364 | 74.912 |  | -.966 | .335 | -219.930 | 75.202 |  |  |
|  | **Age** | -.496 | .249 | -.125 | -1.993 | .047 | -.986 | -.006 | .841 | 1.189 |
|  | **SEX: 1M, 2F** | -1.571 | 6.667 | -.015 | -.236 | .814 | -14.703 | 11.562 | .806 | 1.241 |
|  | **SBP** | -.104 | .175 | -.036 | -.592 | .555 | -.448 | .241 | .898 | 1.114 |
|  | **BMI** (Log) | 69.217 | 44.097 | .098 | 1.570 | .118 | -17.647 | 156.082 | .847 | 1.181 |
|  | **PAC** (Log) | 6.647 | 14.563 | .027 | .456 | .649 | -22.041 | 35.335 | .967 | 1.034 |
|  | **uNa24h** (Square root) | 3.968 | 1.306 | .208 | 3.038 | .003 | 1.395 | 6.541 | .707 | 1.414 |
|  | **uK24h** (Square root) | 9.623 | 2.457 | .255 | 3.917 | .000 | 4.783 | 14.462 | .780 | 1.282 |

Cr Clearance = Creatinine Clearance (ml/min); SEX:1M,2F = 1 male, 2 female; SBP = systolic blood pressure; BMI = Body mass index (Kg/m^2^); PAC = plasma aldosterone concentration (pmol/l); uNa24h = 24h urinary Na^+^ excretion (mmol/d); uK24h = 24h urinary K^+^ excretion (mmol/d).

# CATECHOLAMINE / METANEPHRINE EXCRETION

Renal sympathetic nervous system activity (RSNA) plays a key role in renal haemodynamics and tubular Na^+^ reabsorption;^26^ as an indirect measure of RSNA in our cohort we evaluated the 24h urinary excretion of catecholamines and their more stable catabolites metanephrines (n = 372 and 628 patients, respectively). Urinary norepinephrine and normetanephrine, but not epinephrine or metanephrines that are predominantly produced by the adrenals, increased with higher Na^+^ intake (p < 0.001 and p = 0.014, respectively; online Supplement). The significant association with 24h-uNa^+^ excretion persisted at multivariate analysis for norepinephrine, but not for the less RSAN-specific normetanephrine, after correction for age, sex, systolic blood pressure, BMI and aldosterone , and for use of calcium channel or α-receptor blockers, included in the model as known confounders(p = 0.027).^27^ These findings support the evidence of a highly reabsorptive state in Na^+^ loaded tubules.

## Figure S6. 24h-urinary Catecholamine / Metanephrine Excretion

24h urinary excretion of Catecholamines (top panels) and metanephrines (bottom panels). Data presented as median and IQR. Dunn’s Post-hoc tests: * p < 0.05 vs Low-Na^+^; # p < 0.05 vs Medium-Na^+^

## Table S7. Norepinephrine (NE) and Sodium Excretion – Correlations

| **Correlations** | | **u-NE24h** | **Age** | **SEX 1M, 2F** | **SBP** | **BMI** | **PAC** | **uNa24h** | **CCB** | **AlfaB** |
| --- | --- | --- | --- | --- | --- | --- | --- | --- | --- | --- |
| Pearson Correlation | **u-NE24h** (Log) | 1.000 | .062 | -.288 | .024 | .316 | .079 | .258 | .214 | .266 |
|  | **Age** | .062 | 1.000 | .099 | .312 | .116 | -.010 | -.081 | .047 | .135 |
|  | **SEX: 1M, 2F** | -.288 | .099 | 1.000 | .035 | -.264 | -.098 | -.357 | -.047 | -.159 |
|  | **SBP** | .024 | .312 | .035 | 1.000 | .022 | .016 | -.089 | -.031 | .215 |
|  | **BMI** (Log) | .316 | .116 | -.264 | .022 | 1.000 | .077 | .310 | .102 | .193 |
|  | **PAC** (Log) | .079 | -.010 | -.098 | .016 | .077 | 1.000 | -.060 | .162 | .071 |
|  | **uNa24h** (Square root) | .258 | -.081 | -.357 | -.089 | .310 | -.060 | 1.000 | .018 | .093 |
|  | **CCB** (1Y2N) | .214 | .047 | -.047 | -.031 | .102 | .162 | .018 | 1.000 | .080 |
|  | **AlphaB** (1Y2N) | .266 | .135 | -.159 | .215 | .193 | .071 | .093 | .080 | 1.000 |
| Sig. (1-tailed) | **u-NE24h** (Log) |  | .115 | .000 | .326 | .000 | .065 | .000 | .000 | .000 |
|  | **Age** | .115 |  | .003 | .000 | .004 | .388 | .012 | .098 | .000 |
|  | **SEX: 1M, 2F** | .000 | .003 |  | .189 | .000 | .003 | .000 | .095 | .000 |
|  | **SBP** | .326 | .000 | .189 |  | .314 | .343 | .012 | .218 | .000 |
|  | **BMI** (Log) | .000 | .004 | .000 | .314 |  | .038 | .000 | .009 | .000 |
|  | **PAC** (Log) | .065 | .388 | .003 | .343 | .038 |  | .049 | .000 | .024 |
|  | **uNa24h** (Square root) | .000 | .012 | .000 | .012 | .000 | .049 |  | .314 | .005 |
|  | **CCB** (1Y2N) | .000 | .098 | .095 | .218 | .009 | .000 | .314 |  | .013 |
|  | **AlphaB** (1Y2N) | .000 | .000 | .000 | .000 | .000 | .024 | .005 | .013 |  |
| N | **u-NE24h** (Log) | 372 | 372 | 372 | 345 | 288 | 372 | 372 | 372 | 372 |
|  | **Age** | 372 | 766 | 766 | 647 | 537 | 766 | 766 | 766 | 766 |
|  | **SEX: 1M, 2F** | 372 | 766 | 766 | 647 | 537 | 766 | 766 | 766 | 766 |
|  | **SBP** | 345 | 647 | 647 | 647 | 483 | 647 | 647 | 647 | 647 |
|  | **BMI** (Log) | 288 | 537 | 537 | 483 | 537 | 537 | 537 | 537 | 537 |
|  | **PAC** (Log) | 372 | 766 | 766 | 647 | 537 | 766 | 766 | 766 | 766 |
|  | **uNa24h** (Square root) | 372 | 766 | 766 | 647 | 537 | 766 | 766 | 766 | 766 |
|  | **CCB** (1Y2N) | 372 | 766 | 766 | 647 | 537 | 766 | 766 | 766 | 766 |
|  | **AlphaB** (1Y2N) | 372 | 766 | 766 | 647 | 537 | 766 | 766 | 766 | 766 |

u-NE24h = 24h urinary Norepinephrine excretion (μmol/d); SEX:1M,2F = 1 male, 2 female; SBP = systolic blood pressure; BMI = Body mass index (Kg/m^2^); PAC = plasma aldosterone concentration (pmol/l); uNa24h = 24h urinary Na^+^ excretion (mmol/d); CCB = calcium channel blockers - 1 yes, 2 no; AlphaB = α-blockers - 1 yes, 2 no.

## Table S8. Norepinephrine (NE) and Sodium Excretion – Multivariable regression

| **Model**  Dependent variable: **u-NE24h** | | **Unstandardized Coefficients** | | **Standardized Coefficients** | **t** | **Sig.** | **95.0% Confidence Interval for B** | | **Collinearity Statistics** | |
| --- | --- | --- | --- | --- | --- | --- | --- | --- | --- | --- |
|  |  | **B** | **Std. Error** | **Beta** |  |  | **Lower Bound** | **Upper Bound** | **Tolerance** | **VIF** |
| R^2^ = .16 | (Constant) | 1.161 | .346 |  | 3.357 | .001 | .480 | 1.842 |  |  |
|  | **Age** | .001 | .001 | .062 | 1.060 | .290 | -.001 | .003 | .872 | 1.147 |
|  | **SEX: 1M, 2F** | -.094 | .031 | -.184 | -3.072 | .002 | -.154 | -.034 | .827 | 1.209 |
|  | **SBP** | .000 | .001 | .018 | .312 | .755 | -.001 | .002 | .898 | 1.114 |
|  | **BMI** (Log) | .738 | .205 | .213 | 3.594 | .000 | .334 | 1.141 | .847 | 1.181 |
|  | **PAC** (Log) | .065 | .068 | .053 | .955 | .340 | -.068 | .198 | .973 | 1.028 |
|  | **uNa24h** (Square root) | .013 | .006 | .136 | 2.235 | .026 | .002 | .024 | .801 | 1.249 |
| R^2^ = .22 | (Constant) | 1.432 | .341 |  | 4.198 | .000 | .761 | 2.104 |  |  |
|  | **Age** | .001 | .001 | .040 | .698 | .486 | -.001 | .003 | .865 | 1.156 |
|  | **SEX: 1M, 2F** | -.082 | .030 | -.160 | -2.741 | .007 | -.141 | -.023 | .816 | 1.226 |
|  | **SBP** | .000 | .001 | -.008 | -.133 | .894 | -.002 | .001 | .862 | 1.161 |
|  | **BMI** (Log) | .609 | .201 | .176 | 3.033 | .003 | .214 | 1.004 | .829 | 1.206 |
|  | **PAC** (Log) | .023 | .066 | .018 | .340 | .734 | -.108 | .153 | .948 | 1.054 |
|  | **uNa24h** (Square root) | .012 | .006 | .131 | 2.217 | .027 | .001 | .023 | .800 | 1.250 |
|  | **CCB** (1Y2N) | .086 | .028 | .167 | 3.087 | .002 | .031 | .140 | .958 | 1.044 |
|  | **AlphaB** (1Y2N) | .106 | .034 | .176 | 3.140 | .002 | .040 | .173 | .893 | 1.119 |

u-NE24h = 24h urinary Norepinephrine excretion (μmol/d); SEX:1M,2F = 1 male, 2 female; SBP = systolic blood pressure; BMI = Body mass index (Kg/m^2^); PAC = plasma aldosterone concentration (pmol/l); uNa24h = 24h urinary Na^+^ excretion (mmol/d); CCB = calcium channel blockers - 1 yes, 2 no; AlphaB = α-blockers - 1 yes, 2 no.

## Table S9. Normetanephrine (NM) and Sodium Excretion – Correlations

| **Correlations** | | **u-NM24h** | **Age** | **SEX 1M, 2F** | **SBP** | **BMI** | **PAC** | **uNa24h** | **CCB** | **ALfaB** |
| --- | --- | --- | --- | --- | --- | --- | --- | --- | --- | --- |
| Pearson Correlation | **u-NM24h** (Log) | 1.000 | .099 | -.179 | .042 | .249 | .072 | .137 | .176 | .260 |
|  | **Age** | .099 | 1.000 | .099 | .312 | .116 | -.010 | -.081 | .047 | .135 |
|  | **SEX: 1M, 2F** | -.179 | .099 | 1.000 | .035 | -.264 | -.098 | -.357 | -.047 | -.159 |
|  | **SBP** | .042 | .312 | .035 | 1.000 | .022 | .016 | -.089 | -.031 | .215 |
|  | **BMI** (Log) | .249 | .116 | -.264 | .022 | 1.000 | .077 | .310 | .102 | .193 |
|  | **PAC** (Log) | .072 | -.010 | -.098 | .016 | .077 | 1.000 | -.060 | .162 | .071 |
|  | **uNa24h** (Square root) | .137 | -.081 | -.357 | -.089 | .310 | -.060 | 1.000 | .018 | .093 |
|  | **CCB** (1Y2N) | .176 | .047 | -.047 | -.031 | .102 | .162 | .018 | 1.000 | .080 |
|  | **AlphaB** (1Y2N) | .260 | .135 | -.159 | .215 | .193 | .071 | .093 | .080 | 1.000 |
| Sig. (1-tailed) | **u-NM24h** (Log) |  | .007 | .000 | .163 | .000 | .035 | .000 | .000 | .000 |
|  | **Age** | .007 |  | .003 | .000 | .004 | .388 | .012 | .098 | .000 |
|  | **SEX: 1M, 2F** | .000 | .003 |  | .189 | .000 | .003 | .000 | .095 | .000 |
|  | **SBP** | .163 | .000 | .189 |  | .314 | .343 | .012 | .218 | .000 |
|  | **BMI** (Log) | .000 | .004 | .000 | .314 |  | .038 | .000 | .009 | .000 |
|  | **PAC** (Log) | .035 | .388 | .003 | .343 | .038 |  | .049 | .000 | .024 |
|  | **uNa24h** (Square root) | .000 | .012 | .000 | .012 | .000 | .049 |  | .314 | .005 |
|  | **CCB** (1Y2N) | .000 | .098 | .095 | .218 | .009 | .000 | .314 |  | .013 |
|  | **AlphaB** (1Y2N) | .000 | .000 | .000 | .000 | .000 | .024 | .005 | .013 |  |
| N | **u-NM24h** (Log) | 628 | 628 | 628 | 550 | 452 | 628 | 628 | 628 | 628 |
|  | **Age** | 628 | 766 | 766 | 647 | 537 | 766 | 766 | 766 | 766 |
|  | **SEX: 1M, 2F** | 628 | 766 | 766 | 647 | 537 | 766 | 766 | 766 | 766 |
|  | **SBP** | 550 | 647 | 647 | 647 | 483 | 647 | 647 | 647 | 647 |
|  | **BMI** (Log) | 452 | 537 | 537 | 483 | 537 | 537 | 537 | 537 | 537 |
|  | **PAC** (Log) | 628 | 766 | 766 | 647 | 537 | 766 | 766 | 766 | 766 |
|  | **uNa24h** (Square root) | 628 | 766 | 766 | 647 | 537 | 766 | 766 | 766 | 766 |
|  | **CCB** (1Y2N) | 628 | 766 | 766 | 647 | 537 | 766 | 766 | 766 | 766 |
|  | **AlphaB** (1Y2N) | 628 | 766 | 766 | 647 | 537 | 766 | 766 | 766 | 766 |

u-NM24h = 24h urinary Normetanephrine excretion (μmol/d); SEX:1M,2F = 1 male, 2 female; SBP = systolic blood pressure; BMI = Body mass index (Kg/m^2^); PAC = plasma aldosterone concentration (pmol/l); uNa24h = 24h urinary Na^+^ excretion (mmol/d); CCB = calcium channel blockers - 1 yes, 2 no; AlphaB = α-blockers - 1 yes, 2 no.

## Table S10. Normetanephrine (NM) and Sodium Excretion – Multivariable regression

| **Model**  Dependent variable: **u-NM24h** | | **Unstandardized Coefficients** | | **Standardized Coefficients** | **t** | **Sig.** | **95.0% Confidence Interval for B** | | **Collinearity Statistics** | |
| --- | --- | --- | --- | --- | --- | --- | --- | --- | --- | --- |
|  |  | **B** | **Std. Error** | **Beta** |  |  | **Lower Bound** | **Upper Bound** | **Tolerance** | **VIF** |
| R^2^ = .09 | (Constant) | -1.913 | .343 |  | -5.574 | .000 | -2.587 | -1.238 |  |  |
|  | **Age** | .002 | .001 | .088 | 1.806 | .072 | .000 | .004 | .872 | 1.147 |
|  | **SEX: 1M, 2F** | -.071 | .030 | -.117 | -2.348 | .019 | -.131 | -.012 | .827 | 1.209 |
|  | **SBP** | .000 | .001 | .018 | .378 | .706 | -.001 | .002 | .898 | 1.114 |
|  | **BMI** (Log) | .779 | .203 | .188 | 3.830 | .000 | .379 | 1.179 | .847 | 1.181 |
|  | **PAC** (Log) | .073 | .067 | .050 | 1.086 | .278 | -.059 | .204 | .973 | 1.028 |
|  | **uNa24h** (Square root) | .005 | .006 | .048 | .952 | .342 | -.006 | .017 | .801 | 1.249 |
| R^2^ = .14 | (Constant) | -1.583 | .340 |  | -4.659 | .000 | -2.250 | -.915 |  |  |
|  | **Age** | .002 | .001 | .066 | 1.389 | .165 | -.001 | .004 | .865 | 1.156 |
|  | **SEX: 1M, 2F** | -.056 | .030 | -.091 | -1.871 | .062 | -.114 | .003 | .816 | 1.226 |
|  | **SBP** | .000 | .001 | -.013 | -.272 | .786 | -.002 | .001 | .862 | 1.161 |
|  | **BMI** (Log) | .627 | .200 | .152 | 3.133 | .002 | .234 | 1.020 | .829 | 1.206 |
|  | **PAC** (Log) | .029 | .066 | .020 | .435 | .664 | -.101 | .158 | .948 | 1.054 |
|  | **uNa24h** (Square root) | .005 | .006 | .042 | .856 | .393 | -.006 | .016 | .800 | 1.250 |
|  | **CCB** (1Y2N) | .082 | .028 | .134 | 2.973 | .003 | .028 | .136 | .958 | 1.044 |
|  | **AlphaB** (1Y2N) | .141 | .034 | .194 | 4.176 | .000 | .075 | .207 | .893 | 1.119 |

u-NM24h = 24h urinary Normetanephrine excretion (μmol/d); SEX:1M,2F = 1 male, 2 female; SBP = systolic blood pressure; BMI = Body mass index (Kg/m^2^); PAC = plasma aldosterone concentration (pmol/l); uNa24h = 24h urinary Na^+^ excretion (mmol/d); CCB = calcium channel blockers - 1 yes, 2 no; AlphaB = α-blockers - 1 yes, 2 no.

# METABOLOMICS

## Table S11.1. Peaks: High Na^+^ > Low Na^+^

|  | FC | p | pcorrected | Ion | Evidence | Metabolite | Notes |
| --- | --- | --- | --- | --- | --- | --- | --- |
| **intermediates or end products of the urea cycle / protein catabolism** | 1.39 | 0.0001 | 0.016 | - | Identified +F | **proline** | Conditionally-essential aminoacid; critical for muscle and connective tissues |
|  | 1.52 | 0.0002 | 0.023 | - | Annotated | **n-ac-l-glutamate 5-semialdehyde** | Intermediate in urea cycle |
|  | 1.48 | 0.0002 | 0.023 | - | Identified +F | **Leucine** | Essential, branched-chain aminoacid |
|  | 1.38 | 0.0004 | 0.024 | + | Identified +F | **proline** | Conditionally-essential aminoacid; critical for muscle and connective tissues |
|  | 1.69 | 0.0004 | 0.024 | + | Annotated | **hydroxyprolyl-valine** | an incomplete breakdown product of protein digestion or protein catabolism. |
|  | 1.77 | 0.0004 | 0.024 | + | Annotated | **n6,n6,n6-trimethyl-l-lysine** | methylated derivative of the amino acid lysine; component of histone proteins, a precursor of carnitine and a coenzyme of fatty acid oxidation |
|  | 1.45 | 0.0005 | 0.024 | + | Identified +F | **l-tryptophan** | Essential aminoacid |
|  | 1.44 | 0.0006 | 0.029 | - | Identified | **l-valine** | Essential, branched-chain aminoacid |
|  | 1.48 | 0.0009 | 0.037 | + | Identified +F | **Leucine** | Essential, branched-chain aminoacid |
|  | 1.88 | 0.0013 | 0.043 | + | Annotated | **pyrrolidonecarboxylic acid** | cyclic derivative of glutamic acid |
|  | 1.40 | 0.0017 | 0.048 | - | Identified +F | **tyrosine** | Conditionally-essential aminoacid |
|  | 1.57 | 0.0017 | 0.048 | + | Annotated | **homoarginine** | L-alpha-amino acid |
|  | 1.45 | 0.0018 | 0.050 | + | Identified | **isoleucine** | Essential, branched-chain aminoacid |
|  | 1.54 | 0.0048 | 0.096 | + | Identified | hydroxyproline | major component of the protein collagen; marker of collagen catabolism, tissue degradation or for the consumption of processed meat. |
|  | 1.30 | 0.0054 | 0.106 | + | Annotated | methionyl-cysteine | incomplete breakdown product of protein digestion or protein catabolism. |
|  | 1.33 | 0.006 | 0.108 | - | Annotated | hydroxyproline | major component of the protein collagen; marker of collagen catabolism, tissue degradation or for the consumption of processed meat. |
|  | 1.29 | 0.0062 | 0.108 | + | Annotated | Histidinyl-Cysteine | incomplete breakdown product of protein digestion or protein catabolism. |
|  | 1.39 | 0.0063 | 0.108 | + | Identified +F | l-tyrosine | Conditionally-essential aminoacid |
|  | 1.48 | 0.0068 | 0.109 | + | Annotated | proline | Conditionally-essential aminoacid; critical for muscle and connective tissues |
|  | 1.25 | 0.0069 | 0.109 | - | Identified +F | lysine | Essential aminoacid |
|  | 1.38 | 0.0069 | 0.109 | + | Identified | Lysine | Essential aminoacid |
|  | 1.31 | 0.0072 | 0.110 | + | Annotated | indoleacetic acid | breakdown product of tryptophan metabolism |
|  | **FC** | **p** | **p_corrected_** | **Ion** | **Evidence** | **Metabolite** | **Notes** |
| **urea cycle / protein catabolism** | 1.34 | 0.0095 | 0.125 | - | Annotated | l-phenylalanine | Essential aminoacid |
|  | 1.29 | 0.0126 | 0.149 | + | Identified +F | Threonine | Essential aminoacid |
|  | 1.32 | 0.019 | 0.168 | + | Annotated | citrulline | Intermediate in urea cycle |
|  | 1.18 | 0.02 | 0.170 | - | Annotated | threonine | Essential aminoacid |
|  | 1.26 | 0.0212 | 0.173 | + | Annotated +F | guanidinoacetic acid | metabolite in the Urea cycle and metabolism of amino groups, and in the metabolic pathways of several amino acids. |
|  | 1.53 | 0.0212 | 0.173 | + | Annotated | hydroxyprolyl-histidine | incomplete breakdown product of protein digestion or protein catabolism. |
|  | 1.33 | 0.0238 | 0.186 | - | Annotated | pyroglutamylglycine | alpha amino acid |
|  | 1.21 | 0.0315 | 0.2 | + | Annotated | 3-methylhistidine | product of peptide bond synthesis and methylation of actin and myosin; marker of muscle protein breakdown. |
|  | 1.21 | 0.0374 | 0.231 | + | Annotated | hydantoin-5-propionic acid | metabolite of histidine |
|  | 1.27 | 0.0377 | 0.231 | - | Annotated +F | histidine | Essential aminoacid |
|  | 1.26 | 0.042 | 0.248 | + | Annotated +F | histidine | Essential aminoacid |
|  | 1.24 | 0.0452 | 0.255 | + | Annotated | leucyl-proline | incomplete breakdown product of protein digestion or protein catabolism |
|  | 1.26 | 0.0464 | 0.255 | - | Identified | Ornithine | Intermediate in urea cycle |
|  | 1.18 | 0.0468 | 0.255 | - | Identified | Alanine | Non-essential aminoacid |
| **triglycerides / fatty acid metabolism** | 2.01 | 0.0001 | 0.016 | - | Annotated | **DG** | diacylglycerol |
|  | 2.25 | 0.0001 | 0.016 | + | Annotated | **DG** | diacylglycerol |
|  | 1.39 | 0.0003 | 0.024 | - | Annotated | **Tiglylglycine** | an acyl glycine (minor metabolite of fatty acids). |
|  | 1.21 | 0.0014 | 0.045 | - | Annotated | **undecanoic acid** | medium chain length monocarboxylic acid |
|  | 1.49 | 0.0019 | 0.052 | - | Annotated | 7z,10z-hexadecadienoic acid | fatty acid metabolite |
|  | 1.37 | 0.0043 | 0.09 | - | Annotated | n-undecanoylglycine | acylglycine, with C-11 fatty acid group as the acyl moiety |
|  | 1.39 | 0.01 | 0.128 | + | Annotated | pc(20:4(5z,8z,11z,14z)/0:0) | phosphatidylcholine |
|  | 1.21 | 0.0147 | 0.157 | + | Annotated | pc(18:0/0:0) | glycerophospholipid |
|  | 1.43 | 0.0149 | 0.157 | - | Annotated | 9-keto stearic acid | also called octadecanoic acid; it is one of the useful types of saturated fatty acid that comes from many animal and vegTable S fats and oils |
|  | 1.35 | 0.0158 | 0.159 | + | Annotated | pe(20:4(5z,8z,11z,14z)/0:0) | glycerophospholipid |
|  | 1.35 | 0.0191 | 0.168 | - | Annotated | pe(20:4(5z,8z,11z,14z)/0:0) | glycerophospholipid |
|  | 1.42 | 0.0247 | 0.189 | + | Identified | choline | methyl donor in various metabolic processes, and in lipid metabolism. Choline is now considered to be an essential vitamin. |
|  | **FC** | **p** | **p_corrected_** | **Ion** | **Evidence** | **Metabolite** | **Notes** |
| **TG/FA** | 1.22 | 0.0263 | 0.195 | + | Annotated | pc(o-8:0/o-8:0) | phosphatidylcholine |
|  | 1.37 | 0.0314 | 0.212 | - | Annotated | pe(o-18:1(9z)/0:0) | glycerophospholipid |
|  | 1.21 | 0.034 | 0.222 | + | Annotated | lysopc(18:2(9z,12z)) | lyso-phosphatidylcholine |
|  | 1.68 | 0.0385 | 0.25 | - | Annotated | n-butyrylglycine | an acyl glycine (minor metabolite of fatty acids). |
|  | 1.29 | 0.0465 | 0.255 | - | Annotated | pe(18:1(9z)/0:0) | glycerophospholipid |
| **acyl-carnitines** | 1.68 | 0.0003 | 0.024 | + | Annotated | **valerylcarnitine** | acyl carnitines: organic compounds containing a fatty acid attached to carnitine. They facilitate the transfer of long-chain fatty acids from cytoplasm into mitochondria to undergo beta-oxidation |
|  | 1.78 | 0.0004 | 0.024 | + | Annotated | **isovalerylcarnitine** |  |
|  | 1.84 | 0.0004 | 0.024 | + | Annotated | **isovalerylcarnitine** |  |
|  | 1.47 | 0.0038 | 0.081 | + | Annotated | hydroxyisovaleroyl carnitine |  |
|  | 1.30 | 0.0178 | 0.165 | + | Annotated | palmitoylcarnitine |  |
|  | 1.32 | 0.0419 | 0.248 | + | Annotated | stearoylcarnitine |  |
|  | 1.45 | 0.0463 | 0.255 | + | Annotated | butyrylcarnitine |  |
| **food-related compounds** | 1.40 | 0.0001 | 0.016 | + | Annotated | **pyrrolidine** | in alcoholic beverages and tobacco |
|  | 1.52 | 0.0011 | 0.04 | + | Annotated | **tetramethoxyflavone** | found in herbs and spices |
|  | 1.30 | 0.0024 | 0.062 | + | Annotated | isocrotonic acid | food preservative |
|  | 1.32 | 0.0044 | 0.091 | + | Annotated | 2-napthyloxyacetic acid | food component |
|  | 1.32 | 0.0047 | 0.096 | + | Annotated | tricyclazole | Rice fungicide |
|  | 1.31 | 0.0069 | 0.109 | + | Annotated | dimethylbenzyl carbinyl hexanoate | food additive |
|  | 1.43 | 0.0069 | 0.109 | - | Annotated | 4-methoxybenzyl formate | food flavouring |
|  | 1.24 | 0.0078 | 0.111 | - | Annotated | 4-methylnonanoic acid | flavouring in cooked meats |
|  | 1.31 | 0.0078 | 0.111 | + | Annotated | dihydrochalcone | in mushrooms |
|  | 1.38 | 0.008 | 0.111 | + | Annotated | isocrotonic acid | food preservative |
|  | 1.47 | 0.0087 | 0.119 | + | Annotated | 3,6,8,4',5'-pentamethoxyflavone | found in citrus |
|  | 1.15 | 0.0138 | 0.157 | - | Annotated | phytanic acid | fatty acid (from dairy products, ruminant animal fats, and certain fish) |
|  | 1.33 | 0.0141 | 0.157 | + | Annotated | acetophenone | food flavouring |
|  | 1.31 | 0.0149 | 0.157 | + | Annotated | benzene | used in pesticides and rubbers |
|  | 1.41 | 0.0183 | 0.165 | + | Annotated | (1s,2s,4r,8r)-p-menthane-1,2,8,9-tetrol | found in fats and oils |
|  | **FC** | **p** | **p_corrected_** | **Ion** | **Evidence** | **Metabolite** | **Notes** |
| **food** | 1.32 | 0.0198 | 0.17 | - | Annotated | cyclohexyl 3-methylbutanoate | food flavouring |
|  | 1.17 | 0.0211 | 0.173 | + | Annotated | peperinic acid | in aged peppermint oil. |
|  | 1.52 | 0.0212 | 0.173 | + | Annotated | geniposidic acid | in beverages |
|  | 1.43 | 0.022 | 0.173 | - | Annotated | 1-OH-1-phenyl-3-hexadecanone | found in fats and oils. |
|  | 1.35 | 0.0257 | 0.193 | + | Annotated | 3-(2-furanyl)-2-phenyl-2-propenal | flavouring for tobacco and food product |
|  | 1.22 | 0.0266 | 0.195 | - | Annotated | ethyl (±)-3-methylpentanoate | flavouring ingredient |
|  | 1.38 | 0.0424 | 0.248 | - | Annotated | 1-phenyl-1,3-hexadecanedione | in fats and oils |
| **Others** | 2.38 | 0.0002 | 0.023 | - | Annotated | **DHEAs** | Sex hormone |
|  | 1.55 | 0.0258 | 0.193 | - | Annotated | **Androsterone sulfate** | Sex hormone |
|  | 1.72 | 0.0005 | 0.024 | - | Annotated | **1alpha,25-dihydroxy (…) vit d3** | Vit D derivative |
|  | 1.46 | 0.0006 | 0.029 | - | Annotated | **3r-hydroxy-hexanoic acid** | component of urine |
|  | 1.52 | 0.0026 | 0.064 | - | Identified | rhamnose | 6-deoxy-hexose |
|  | 1.64 | 0.0036 | 0.078 | + | Annotated | dihydrothymine | intermediate breakdown product of thymine; toxic at high levels |
|  | 1.39 | 0.0061 | 0.108 | - | Annotated | 3-hydroxy-2-methylpyridine-4,5-dicarboxylate | last step in the synthesis of succinate semialdehyde, an intermediate in butanoate metabolism. |
|  | 1.32 | 0.0077 | 0.111 | + | Annotated | 1alpha,25-dihydroxy (…) vit d3 | Vit D derivative |
|  | 1.30 | 0.0093 | 0.124 | - | Annotated | 5-hydroxyindoleacetic acid | breakdown product of serotonin that is excreted in the urine. |
|  | 1.24 | 0.0162 | 0.159 | + | Annotated | xanthylic acid | metabolic intermediate in the Purine Metabolism |
|  | 1.40 | 0.0163 | 0.159 | - | Annotated | cholesterol sulfate | associated with hypercholesterolemia |
|  | 1.21 | 0.0273 | 0.198 | + | Identified +F | creatinine | - |
|  | 1.33 | 0.0277 | 0.198 | - | Annotated | (r)-3-hydroxydecanoic acid | intermediate in fatty acid biosynthesis. |
|  | 1.42 | 0.0318 | 0.212 | + | Annotated | Noradrenaline | - |
|  | 1.21 | 0.0321 | 0.215 | - | Annotated | glucosamine | aminosugar |
|  | 1.30 | 0.0455 | 0.255 | - | Annotated | n1-met-2-pyridone-5-carboxamide | uremic toxin. |
|  | 1.34 | 0.0463 | 0.255 | + | Annotated | n1-met-2-pyridone-5-carboxamide | uremic toxin. |
|  | 1.33 | 0.0469 | 0.255 | + | Annotated | Lipoic acid | Enzymatic cofactor (e.g. citric acid cycle) |

FC = fold change; p and p_corrected_ (see statistics in methods) High-Na^+^ vs Low-Na+, p_corrected_< 0.05 in bold and dark grey shaded. Ion: positive or negative mode peak acquisition. Evidence for LC/MS peaks: identified (matched by retention time and mass to a standard) or annotated (assigned putatively on the basis of mass) compounds ± fragmentation (F), based on the Metabolite Standards Initiative guidelines.^28^

## Table S11.2. Peaks: High Na^+^ < Low Na^+^.

|  | FC | p | p_corrected_ | Ion | Evidence | Metabolite | Notes |
| --- | --- | --- | --- | --- | --- | --- | --- |
| **uc/pc** | 0,74 | 0,0124 | 0,149 | + | Annotated | alanyl-aspartate | incomplete breakdown product of protein digestion/catabolism. |
|  | 0,75 | 0,0161 | 0,159 | - | Annotated | alanyl-aspartate | incomplete breakdown product of protein digestion/catabolism. |
|  | 0,60 | 0,0247 | 0,189 | - | Annotated | glycyl-arginine | incomplete breakdown product of protein digestion/catabolism. |
|  | 0,76 | 0,0360 | 0,227 | + | Annotated | prolyl-glutamate | incomplete breakdown product of protein digestion/catabolism. |
| **food** | 0,65 | 0,0034 | 0,076 | - | Annotated | 3-hydroxyphenylacetic acid | Antioxidant. It has a protective biological activity in human. |
|  | 0,71 | 0,0056 | 0,106 | - | Annotated | cyclohexyl 3-methylbutanoate | food flavouring (blackcurrant) |
|  | 0,80 | 0,0113 | 0,141 | - | Annotated | beta-d-3-[5-deoxy-5-(dimethylarsinyl)ribofuranosyloxy]-2-hydroxy-1-propanesulfonic acid | found in green vegTable Ss |
|  | 0,82 | 0,0153 | 0,159 | + | Annotated | (2alpha,3alpha,5alpha,22r,23r)-2,3,22,23-tetrahydroxy-25-methylergost-24(28)en-6-one | found in common bean |
|  | 0,76 | 0,0427 | 0,249 | - | Annotated | wharangin | found in green vegTable Ss |
| **Others** | 0,78 | 0,0154 | 0,159 | + | Annotated | 4-Amino-3-hydroxybutyrate | hydroxy fatty acid |
|  | 0,76 | 0,0197 | 0,170 | - | Annotated | l-aspartate 4-semialdehyde | involved in lysine / homoserine biosynthesis |
|  | 0,84 | 0,0359 | 0,228 | + | Annotated | n-acetyl-l-aspartate | source of acetate for lipid, myelin and CNS neurotransmitters. |

Uc/pc = urea cycle/protein catabolism. FC = fold change; p and p_corrected_ (see statistics in methods) High-Na^+^ vs Low-Na*. Ion: positive or negative mode peak acquisition. Evidence for LC/MS peaks: identified (matched by retention time and mass to a standard) or annotated (assigned putatively on the basis of mass) compounds ± fragmentation (F), based on the Metabolite Standards Initiative guidelines.^28^

# GLUCIDIC METABOLISM

## Table S12. Glucose and Sodium Excretion – Correlations

| **Correlations** | | **Glucose** | **Age** | **SEX 1M, 2F** | **SBP** | **BMI** | **PAC** | **uNa24h** |
| --- | --- | --- | --- | --- | --- | --- | --- | --- |
| Pearson Correlation | **Glucose** | 1.000 | .319 | -.283 | .072 | .357 | .133 | .179 |
|  | **Age** | .319 | 1.000 | .099 | .312 | .116 | -.010 | -.081 |
|  | **SEX: 1M, 2F** | -.283 | .099 | 1.000 | .035 | -.264 | -.098 | -.357 |
|  | **SBP** | .072 | .312 | .035 | 1.000 | .022 | .016 | -.089 |
|  | **BMI** (Log) | .357 | .116 | -.264 | .022 | 1.000 | .077 | .310 |
|  | **PAC** (Log) | .133 | -.010 | -.098 | .016 | .077 | 1.000 | -.060 |
|  | **uNa24h** (Square root) | .179 | -.081 | -.357 | -.089 | .310 | -.060 | 1.000 |
| Sig. (1-tailed) | **Glucose** |  | .000 | .000 | .203 | .000 | .041 | .010 |
|  | **Age** | .000 |  | .003 | .000 | .004 | .388 | .012 |
|  | **SEX: 1M, 2F** | .000 | .003 |  | .189 | .000 | .003 | .000 |
|  | **SBP** | .203 | .000 | .189 |  | .314 | .343 | .012 |
|  | **BMI** (Log) | .000 | .004 | .000 | .314 |  | .038 | .000 |
|  | **PAC** (Log) | .041 | .388 | .003 | .343 | .038 |  | .049 |
|  | **uNa24h** (Square root) | .010 | .012 | .000 | .012 | .000 | .049 |  |
| N | **Glucose** | 171 | 171 | 171 | 136 | 127 | 171 | 171 |
|  | **Age** | 171 | 766 | 766 | 647 | 537 | 766 | 766 |
|  | **SEX: 1M, 2F** | 171 | 766 | 766 | 647 | 537 | 766 | 766 |
|  | **SBP** | 136 | 647 | 647 | 647 | 483 | 647 | 647 |
|  | **BMI** (Log) | 127 | 537 | 537 | 483 | 537 | 537 | 537 |
|  | **PAC** (Log) | 171 | 766 | 766 | 647 | 537 | 766 | 766 |
|  | **uNa24h** (Square root) | 171 | 766 | 766 | 647 | 537 | 766 | 766 |

Glucose = plasma fasting glucose (mmol/l); SEX:1M,2F = 1 male, 2 female; SBP = systolic blood pressure; BMI = Body mass index (Kg/m^2^); PAC = plasma aldosterone concentration (pmol/l); uNa24h = 24h urinary Na^+^ excretion (mmol/d).

## Table S13. Insulin and Sodium Excretion – Correlations

| **Correlations** | | **Insulin** (Log) | **Age** | **SEX 1M, 2F** | **SBP** | **BMI** | **PAC** | **uNa24h** |
| --- | --- | --- | --- | --- | --- | --- | --- | --- |
| Pearson Correlation | **Insulin** (Log) | 1.000 | -.009 | -.182 | -.042 | .478 | .031 | .177 |
|  | **Age** | -.009 | 1.000 | .099 | .312 | .116 | -.010 | -.081 |
|  | **SEX: 1M, 2F** | -.182 | .099 | 1.000 | .035 | -.264 | -.098 | -.357 |
|  | **SBP** | -.042 | .312 | .035 | 1.000 | .022 | .016 | -.089 |
|  | **BMI** (Log) | .478 | .116 | -.264 | .022 | 1.000 | .077 | .310 |
|  | **PAC** (Log) | .031 | -.010 | -.098 | .016 | .077 | 1.000 | -.060 |
|  | **uNa24h** (Square root) | .177 | -.081 | -.357 | -.089 | .310 | -.060 | 1.000 |
| Sig. (1-tailed) | **Insulin** (Log) |  | .426 | .000 | .210 | .000 | .258 | .000 |
|  | **Age** | .426 |  | .003 | .000 | .004 | .388 | .012 |
|  | **SEX: 1M, 2F** | .000 | .003 |  | .189 | .000 | .003 | .000 |
|  | **SBP** | .210 | .000 | .189 |  | .314 | .343 | .012 |
|  | **BMI** (Log) | .000 | .004 | .000 | .314 |  | .038 | .000 |
|  | **PAC** (Log) | .258 | .388 | .003 | .343 | .038 |  | .049 |
|  | **uNa24h** (Square root) | .000 | .012 | .000 | .012 | .000 | .049 |  |
| N | **Insulin** (Log) | 447 | 447 | 447 | 369 | 292 | 447 | 447 |
|  | **Age** | 447 | 766 | 766 | 647 | 537 | 766 | 766 |
|  | **SEX: 1M, 2F** | 447 | 766 | 766 | 647 | 537 | 766 | 766 |
|  | **SBP** | 369 | 647 | 647 | 647 | 483 | 647 | 647 |
|  | **BMI** (Log) | 292 | 537 | 537 | 483 | 537 | 537 | 537 |
|  | **PAC** (Log) | 447 | 766 | 766 | 647 | 537 | 766 | 766 |
|  | **uNa24h** (Square root) | 447 | 766 | 766 | 647 | 537 | 766 | 766 |

Insulin = plasma fasting insulin (μU/ml); SEX:1M,2F = 1 male, 2 female; SBP = systolic blood pressure; BMI = Body mass index (Kg/m^2^); PAC = plasma aldosterone concentration (pmol/l); uNa24h = 24h urinary Na^+^ excretion (mmol/d).

**Table S14. Glucose and Sodium Excretion – Multivariable regression**

| **Model**  Dependent variable: **Glucose** | | **Unstandardized Coefficients** | | **Standardized Coefficients** | **t** | **Sig.** | **95.0% Confidence Interval for B** | | **Collinearity Statistics** | |
| --- | --- | --- | --- | --- | --- | --- | --- | --- | --- | --- |
|  |  | **B** | **Std. Error** | **Beta** |  |  | **Lower Bound** | **Upper Bound** | **Tolerance** | **VIF** |
| R^2^ = .24 | (Constant) | .628 | 1.295 |  | .485 | .629 | -1.936 | 3.192 |  |  |
|  | **Age** | .017 | .004 | .327 | 3.929 | .000 | .008 | .025 | .872 | 1.147 |
|  | **SEX: 1M, 2F** | -.298 | .115 | -.223 | -2.602 | .010 | -.525 | -.071 | .827 | 1.209 |
|  | **SBP** | -.001 | .003 | -.024 | -.296 | .768 | -.007 | .005 | .898 | 1.114 |
|  | **BMI** (Log) | 2.144 | .768 | .236 | 2.791 | .006 | .623 | 3.664 | .847 | 1.181 |
|  | **PAC** (Log) | .322 | .253 | .100 | 1.273 | .205 | -.179 | .823 | .973 | 1.028 |
|  | **uNa24h** (Square root) | .014 | .021 | .057 | .654 | .515 | -.028 | .056 | .801 | 1.249 |

Glucose = plasma fasting glucose (mmol/l); SEX:1M,2F = 1 male, 2 female; SBP = systolic blood pressure; BMI = Body mass index (Kg/m^2^); PAC = plasma aldosterone concentration (pmol/l); uNa24h = 24h urinary Na^+^ excretion (mmol/d).

**Table S15. Insulin and Sodium Excretion – Multivariable regression**

| **Model**  Dependent variable: **Insulin** | | **Unstandardized Coefficients** | | **Standardized Coefficients** | **t** | **Sig.** | **95.0% Confidence Interval for B** | | **Collinearity Statistics** | |
| --- | --- | --- | --- | --- | --- | --- | --- | --- | --- | --- |
|  |  | **B** | **Std. Error** | **Beta** |  |  | **Lower Bound** | **Upper Bound** | **Tolerance** | **VIF** |
| R^2^ = .22 | (Constant) | -1.565 | .363 |  | -4.313 | .000 | -2.279 | -.851 |  |  |
|  | **Age** | -.001 | .001 | -.047 | -.848 | .397 | -.003 | .001 | .872 | 1.147 |
|  | **SEX: 1M, 2F** | -.029 | .032 | -.051 | -.902 | .368 | -.092 | .034 | .827 | 1.209 |
|  | **SBP** | -.001 | .001 | -.035 | -.649 | .517 | -.002 | .001 | .898 | 1.114 |
|  | **BMI** (Log) | 1.800 | .215 | .470 | 8.367 | .000 | 1.377 | 2.224 | .847 | 1.181 |
|  | **PAC** (Log) | -.013 | .071 | -.010 | -.190 | .849 | -.153 | .126 | .973 | 1.028 |
|  | **uNa24h** (Square root) | .000 | .006 | .005 | .080 | .936 | -.011 | .012 | .801 | 1.249 |

Insulin = plasma fasting insulin (μU/ml); SEX:1M,2F = 1 male, 2 female; SBP = systolic blood pressure; BMI = Body mass index (Kg/m^2^); PAC = plasma aldosterone concentration (pmol/l); uNa24h = 24h urinary Na^+^ excretion (mmol/d).

# CORTISOL

## Table S16. Urinary Free Cortisol and Sodium Excretion – Correlations

| **Correlations** | | **UFC 24h** | **Age** | **SEX 1M, 2F** | **SBP** | **BMI** | **PAC** | **uNa24h** | **Cr Clear** |
| --- | --- | --- | --- | --- | --- | --- | --- | --- | --- |
| Pearson Correlation | **UFC 24h** (Log) | 1.000 | .019 | -.239 | -.011 | -.001 | .068 | .391 | .458 |
|  | **Age** | .019 | 1.000 | .099 | .312 | .116 | -.010 | -.081 | -.130 |
|  | **SEX: 1M, 2F** | -.239 | .099 | 1.000 | .035 | -.264 | -.098 | -.357 | -.323 |
|  | **SBP** | -.011 | .312 | .035 | 1.000 | .022 | .016 | -.089 | -.138 |
|  | **BMI** (Log) | -.001 | .116 | -.264 | .022 | 1.000 | .077 | .310 | .412 |
|  | **PAC** (Log) | .068 | -.010 | -.098 | .016 | .077 | 1.000 | -.060 | .059 |
|  | **uNa24h** (Square root) | .391 | -.081 | -.357 | -.089 | .310 | -.060 | 1.000 | .483 |
|  | **Cr Clearance** | .458 | -.130 | -.323 | -.138 | .412 | .059 | .483 | 1.000 |
| Sig. (1-tailed) | **UFC 24h** (Log) | . | .411 | .002 | .450 | .498 | .215 | .000 | .000 |
|  | **Age** | .411 | . | .003 | .000 | .004 | .388 | .012 | .021 |
|  | **SEX: 1M, 2F** | .002 | .003 | . | .189 | .000 | .003 | .000 | .000 |
|  | **SBP** | .450 | .000 | .189 | . | .314 | .343 | .012 | .023 |
|  | **BMI** (Log) | .498 | .004 | .000 | .314 | . | .038 | .000 | .000 |
|  | **PAC** (Log) | .215 | .388 | .003 | .343 | .038 | . | .049 | .180 |
|  | **uNa24h** (Square root) | .000 | .012 | .000 | .012 | .000 | .049 | . | .000 |
|  | **Cr Clearance** | .000 | .021 | .000 | .023 | .000 | .180 | .000 | . |
| N | **UFC 24h** (Log) | 137 | 137 | 137 | 125 | 123 | 137 | 137 | 82 |
|  | **Age** | 137 | 766 | 766 | 647 | 537 | 766 | 766 | 245 |
|  | **SEX: 1M, 2F** | 137 | 766 | 766 | 647 | 537 | 766 | 766 | 245 |
|  | **SBP** | 125 | 647 | 647 | 647 | 483 | 647 | 647 | 211 |
|  | **BMI** (Log) | 123 | 537 | 537 | 483 | 537 | 537 | 537 | 204 |
|  | **PAC** (Log) | 137 | 766 | 766 | 647 | 537 | 766 | 766 | 245 |
|  | **uNa24h** (Square root) | 137 | 766 | 766 | 647 | 537 | 766 | 766 | 245 |
|  | **Cr Clearance** | 82 | 245 | 245 | 211 | 204 | 245 | 245 | 245 |

UFC 24h = 24h urinary free cortisol (nmol/d); SEX:1M,2F = 1 male, 2 female; SBP = systolic blood pressure; BMI = Body mass index (Kg/m^2^); PAC = plasma aldosterone concentration (pmol/l); uNa24h = 24h urinary Na^+^ excretion (mmol/d); Cr Clearance = Creatinine Clearance (ml/min).

## Table S17. Urinary Free Cortisol and Sodium Excretion – Multivariable regression

| **Model**  Dependent variable: **UFC 24h** (Log) | | **Unstandardized Coefficients** | | **Standardized Coefficients** | **t** | **Sig.** | **95.0% Confidence Interval for B** | | **Collinearity Statistics** | |
| --- | --- | --- | --- | --- | --- | --- | --- | --- | --- | --- |
|  |  | **B** | **Std. Error** | **Beta** |  |  | **Lower Bound** | **Upper Bound** | **Tolerance** | **VIF** |
| R^2^ = .20 | (Constant) | 1.948 | 0.437 |  | 4.460 | .000 | 1.083 | 2.813 |  |  |
|  | **Age** | .001 | .001 | .087 | 0.982 | .328 | -.001 | .004 | .872 | 1.147 |
|  | **SEX: 1M, 2F** | -0.059 | 0.039 | -.140 | -1.532 | .128 | -0.136 | 0.017 | .827 | 1.209 |
|  | **SBP** | .000 | .001 | .006 | 0.063 | .950 | -.002 | .002 | .898 | 1.114 |
|  | **BMI** (Log) | -0.525 | 0.259 | -.183 | -2.026 | .045 | -1.038 | -0.012 | .847 | 1.181 |
|  | **PAC** (Log) | 0.095 | 0.085 | .094 | 1.114 | .268 | -0.074 | 0.264 | .973 | 1.028 |
|  | **uNa24h** (Square root) | 0.032 | .007 | .411 | 4.436 | .000 | 0.018 | 0.046 | .801 | 1.249 |
| R^2^ = .34 | (Constant) | 2.265 | 0.506 |  | 4.479 | .000 | 1.257 | 3.272 |  |  |
|  | **Age** | .002 | .002 | .132 | 1.295 | .199 | -.001 | .005 | .860 | 1.162 |
|  | **SEX: 1M, 2F** | -0.039 | 0.044 | -.091 | -.869 | .387 | -0.127 | 0.050 | .815 | 1.227 |
|  | **SBP** | .000 | .001 | .041 | 0.412 | .682 | -.002 | .003 | .890 | 1.123 |
|  | **BMI** (Log) | -0.897 | 0.311 | -.312 | -2.881 | .005 | -1.517 | -0.276 | .766 | 1.306 |
|  | **PAC** (Log) | 0.074 | 0.098 | .073 | .754 | .453 | -0.121 | 0.268 | .970 | 1.031 |
|  | **uNa24h** (Square root) | 0.020 | .009 | .256 | 2.262 | .027 | 0.002 | 0.037 | .700 | 1.429 |
|  | **Cr Clearance** | 0.002 | 0.001 | .452 | 3.858 | .000 | 0.001 | 0.003 | .655 | 1.527 |

UFC 24h = 24h urinary free cortisol (nmol/d); SEX:1M,2F = 1 male, 2 female; SBP = systolic blood pressure; BMI = Body mass index (Kg/m^2^); PAC = plasma aldosterone concentration (pmol/l); uNa24h = 24h urinary Na^+^ excretion (mmol/d); Cr Clearance = Creatinine Clearance (ml/min).

## Table S18. Morning Plasma Cortisol Concentration and Sodium Excretion – Correlations

| **Correlations** | | **PCC** | **Age** | **SEX 1M, 2F** | **SBP** | **BMI** | **PAC** | **uNa24h** | **Cr Clear** |
| --- | --- | --- | --- | --- | --- | --- | --- | --- | --- |
| Pearson Correlation | **PCC** (Log) | 1.000 | -.013 | -.090 | .120 | -.201 | .074 | -.083 | -.060 |
|  | **Age** | -.013 | 1.000 | .099 | .312 | .116 | -.010 | -.081 | -.130 |
|  | **SEX: 1M, 2F** | -.090 | .099 | 1.000 | .035 | -.264 | -.098 | -.357 | -.323 |
|  | **SBP** | .120 | .312 | .035 | 1.000 | .022 | .016 | -.089 | -.138 |
|  | **BMI** (Log) | -.201 | .116 | -.264 | .022 | 1.000 | .077 | .310 | .412 |
|  | **PAC** (Log) | .074 | -.010 | -.098 | .016 | .077 | 1.000 | -.060 | .059 |
|  | **uNa24h** (Square root) | -.083 | -.081 | -.357 | -.089 | .310 | -.060 | 1.000 | .483 |
|  | **Cr Clearance** | -.060 | -.130 | -.323 | -.138 | .412 | .059 | .483 | 1.000 |
| Sig. (1-tailed) | **PCC** | . | .369 | .011 | .002 | .000 | .029 | .017 | .188 |
|  | **Age** | .369 | . | .003 | .000 | .004 | .388 | .012 | .021 |
|  | **SEX: 1M, 2F** | .011 | .003 | . | .189 | .000 | .003 | .000 | .000 |
|  | **SBP** | .002 | .000 | .189 | . | .314 | .343 | .012 | .023 |
|  | **BMI** (Log) | .000 | .004 | .000 | .314 | . | .038 | .000 | .000 |
|  | **PAC** (Log) | .029 | .388 | .003 | .343 | .038 | . | .049 | .180 |
|  | **uNa24h** (Square root) | .017 | .012 | .000 | .012 | .000 | .049 | . | .000 |
|  | **Cr Clearance** | .188 | .021 | .000 | .023 | .000 | .180 | .000 | . |
| N | **PCC** | 658 | 658 | 658 | 549 | 444 | 658 | 658 | 217 |
|  | **Age** | 658 | 766 | 766 | 647 | 537 | 766 | 766 | 245 |
|  | **SEX: 1M, 2F** | 658 | 766 | 766 | 647 | 537 | 766 | 766 | 245 |
|  | **SBP** | 549 | 647 | 647 | 647 | 483 | 647 | 647 | 211 |
|  | **BMI** (Log) | 444 | 537 | 537 | 483 | 537 | 537 | 537 | 204 |
|  | **PAC** (Log) | 658 | 766 | 766 | 647 | 537 | 766 | 766 | 245 |
|  | **uNa24h** (Square root) | 658 | 766 | 766 | 647 | 537 | 766 | 766 | 245 |
|  | **Cr Clearance** | 217 | 245 | 245 | 211 | 204 | 245 | 245 | 245 |

PCC = morning plasma cortisol concentration (nmol/l); SEX:1M,2F = 1 male, 2 female; SBP = systolic blood pressure; BMI = Body mass index (Kg/m^2^); PAC = plasma aldosterone concentration (pmol/l); uNa24h = 24h urinary Na^+^ excretion (mmol/d); Cr Clearance = Creatinine Clearance (ml/min).

## Table S19. Morning Plasma Cortisol Concentration and Sodium Excretion – Multivariable regression

| **Model**  Dependent variable: **PCC** (Log) | | **Unstandardized Coefficients** | | **Standardized Coefficients** | **t** | **Sig.** | **95.0% Confidence Interval for B** | | **Collinearity Statistics** | |
| --- | --- | --- | --- | --- | --- | --- | --- | --- | --- | --- |
|  |  | **B** | **Std. Error** | **Beta** |  |  | **Lower Bound** | **Upper Bound** | **Tolerance** | **VIF** |
| R^2^ = .09 | (Constant) | 2.917 | 0.179 |  | 16.287 | .000 | 2.565 | 3.269 |  |  |
|  | **Age** | .000 | .001 | -.013 | -0.268 | .789 | -.001 | .001 | .872 | 1.147 |
|  | **SEX: 1M, 2F** | -0.053 | 0.016 | -.167 | -3.333 | .001 | -0.084 | -0.022 | .827 | 1.209 |
|  | **SBP** | .001 | .000 | .129 | 2.675 | .008 | .000 | .002 | .898 | 1.114 |
|  | **BMI** (Log) | -0.503 | 0.106 | -.235 | -4.734 | .000 | -0.712 | -0.294 | .847 | 1.181 |
|  | **PAC** (Log) | 0.053 | 0.035 | .070 | 1.511 | .132 | -0.016 | 0.122 | .973 | 1.028 |
|  | **uNa24h** (Square root) | -0.003 | .003 | -.055 | -1.072 | .284 | -0.009 | 0.003 | .801 | 1.249 |
| R^2^ = .09 | (Constant) | 2.934 | 0.271 |  | 10.828 | .000 | 2.399 | 3.468 |  |  |
|  | **Age** | .000 | .001 | -.010 | -0.135 | .892 | -.002 | .002 | .860 | 1.162 |
|  | **SEX: 1M, 2F** | -0.052 | 0.024 | -.164 | -2.172 | .031 | -0.099 | -0.005 | .815 | 1.227 |
|  | **SBP** | .001 | .001 | .131 | 1.819 | .070 | .000 | .002 | .890 | 1.123 |
|  | **BMI** (Log) | -0.522 | 0.167 | -.244 | -3.132 | .002 | -0.851 | -0.193 | .766 | 1.306 |
|  | **PAC** (Log) | 0.052 | 0.052 | .068 | .989 | .324 | -0.051 | 0.155 | .970 | 1.031 |
|  | **uNa24h** (Square root) | -0.004 | .005 | -.066 | -0.805 | .422 | -0.013 | 0.006 | .700 | 1.429 |
|  | **Cr Clearance** | 0.000 | 0.000 | .032 | 0.376 | .707 | -0.001 | 0.001 | .655 | 1.527 |

PCC = morning plasma cortisol concentration (nmol/l); SEX:1M,2F = 1 male, 2 female; SBP = systolic blood pressure; BMI = Body mass index (Kg/m^2^); PAC = plasma aldosterone concentration (pmol/l); uNa24h = 24h urinary Na^+^ excretion (mmol/d); Cr Clearance = Creatinine Clearance (ml/min).

# ONLINE SUPPLEMENT REFERENCES

1. Mancia G, Fagard R, Narkiewicz K, Redon J, Zanchetti A, Bohm M, Christiaens T, Cifkova R, De Backer G, Dominiczak A, et al. 2013 esh/esc guidelines for the management of arterial hypertension: The task force for the management of arterial hypertension of the european society of hypertension (ESH) and of the european society of cardiology (ESC). *European heart journal*. 2013;34:2159-2219

2. Funder JW, Carey RM, Mantero F, Murad MH, Reincke M, Shibata H, Stowasser M, Young WF, Jr. The management of primary aldosteronism: Case detection, diagnosis, and treatment: An endocrine society clinical practice guideline. *The Journal of clinical endocrinology and metabolism*. 2016;101:1889-1916

3. Nieman LK, Biller BM, Findling JW, Newell-Price J, Savage MO, Stewart PM, Montori VM. The diagnosis of cushing's syndrome: An endocrine society clinical practice guideline. *The Journal of clinical endocrinology and metabolism*. 2008;93:1526-1540

4. Lenders JW, Duh QY, Eisenhofer G, Gimenez-Roqueplo AP, Grebe SK, Murad MH, Naruse M, Pacak K, Young WF, Jr. Pheochromocytoma and paraganglioma: An endocrine society clinical practice guideline. *The Journal of clinical endocrinology and metabolism*. 2014;99:1915-1942

5. Levey AS, Stevens LA, Schmid CH, Zhang YL, Castro AF, 3rd, Feldman HI, Kusek JW, Eggers P, Van Lente F, Greene T, Coresh J. A new equation to estimate glomerular filtration rate. *Annals of internal medicine*. 2009;150:604-612

6. Cogswell ME, Loria CM, Terry AL, Zhao L, Wang CY, Chen TC, Wright JD, Pfeiffer CM, Merritt R, Moy CS, Appel LJ. Estimated 24-hour urinary sodium and potassium excretion in us adults. *Jama*. 2018;319:1209-1220

7. Institute of Medicine, Panel on Dietary Reference Intakes for Electrolytes and Water: *Dietary Reference Intakes for Water, Potassium Sodium, Chloride, and Sulfate.* Washington, DC: National Academies Press; 2005.

8. Institute of Medicine. Strategies to Reduce Sodium Intake in the United States. Washington, DC: National Academies Press; 2010.

9. Rossi GP, Ceolotto G, Rossitto G, Seccia TM, Maiolino G, Berton C, Basso D, Plebani M. Prospective validation of an automated chemiluminescence-based assay of renin and aldosterone for the work-up of arterial hypertension. *Clinical chemistry and laboratory medicine*. 2016;54:1441-1450

10. Maiolino G, Rossitto G, Bisogni V, Cesari M, Seccia TM, Plebani M, Rossi GP. Quantitative value of aldosterone-renin ratio for detection of aldosterone-producing adenoma: The aldosterone-renin ratio for primary aldosteronism (aquarr) study. *Journal of the American Heart Association*. 2017;6

11. Antonelli G, Artusi C, Marinova M, Brugnolo L, Zaninotto M, Scaroni C, Gatti R, Mantero F, Plebani M. Cortisol and cortisone ratio in urine: Lc-ms/ms method validation and preliminary clinical application. *Clinical chemistry and laboratory medicine*. 2014;52:213-220

12. Ceccato F, Antonelli G, Barbot M, Zilio M, Mazzai L, Gatti R, Zaninotto M, Mantero F, Boscaro M, Plebani M, Scaroni C. The diagnostic performance of urinary free cortisol is better than the cortisol:Cortisone ratio in detecting de novo cushing's syndrome: The use of a lc-ms/ms method in routine clinical practice. *European journal of endocrinology*. 2014;171:1-7

13. Passing H, Bablok. A new biometrical procedure for testing the equality of measurements from two different analytical methods. Application of linear regression procedures for method comparison studies in clinical chemistry, part i. *Journal of clinical chemistry and clinical biochemistry.* 1983;21:709-720

14. Klahr S, Hamm LL, Hammerman MR, Mandel LJ. Renal metabolism: Integrated responses. *Comprehensive physiology*. 2011:2263-2333.

15. Chambers MC, Maclean B, Burke R, Amodei D, Ruderman DL, Neumann S, Gatto L, Fischer B, Pratt B, Egertson J, et al. P. A cross-platform toolkit for mass spectrometry and proteomics. *Nature biotechnology*. 2012;30:918-920

16. Smith CA, Want EJ, O'Maille G, Abagyan R, Siuzdak G. Xcms: Processing mass spectrometry data for metabolite profiling using nonlinear peak alignment, matching, and identification. *Analytical chemistry*. 2006;78:779-787

17. Scheltema RA, Jankevics A, Jansen RC, Swertz MA, Breitling R. Peakml/mzmatch: A file format, java library, r library, and tool-chain for mass spectrometry data analysis. *Analytical chemistry*. 2011;83:2786-2793

18. Gloaguen Y, Morton F, Daly R, Gurden R, Rogers S, Wandy J, Wilson D, Barrett M, Burgess K. Pimp my metabolome: An integrated, web-based tool for lc-ms metabolomics data. *Bioinformatics*. 2017;33:4007-4009

19. Motulsky HJ, Brown RE. Detecting outliers when fitting data with nonlinear regression - a new method based on robust nonlinear regression and the false discovery rate. *BMC bioinformatics*. 2006;7:123

20. Kanehisa M, Goto S. Kegg: Kyoto encyclopedia of genes and genomes. *Nucleic acids research*. 2000;28:27-30

21. Wishart DS, Feunang YD, Marcu A, Guo AC, Liang K, Vazquez-Fresno R, Sajed T, Johnson D, Li C, Karu N, et al. Hmdb 4.0: The human metabolome database for 2018. *Nucleic acids research*. 2018;46:D608-d617

22. Fahy E, Sud M, Cotter D, Subramaniam S. Lipid maps online tools for lipid research. *Nucleic acids research*. 2007;35:W606-612

23. Ritchie ME, Phipson B, Wu D, Hu Y, Law CW, Shi W, Smyth GK. Limma powers differential expression analyses for rna-sequencing and microarray studies. *Nucleic acids research*. 2015;43:e47

24. Storey JD, Bass AJ, Dabney A, Robinson D. qvalue: Q-value estimation for false discovery rate control. R package version 2.16.0. 2019; <http://github.com/jdstorey/qvalue>

25. Benjamini Y, Krieger AM, D. Y. Adaptive linear step-up procedures that control the false discovery rate. *Biometrika*. 2006;93:491-507

26. Kopp UC. *Neural control of renal function*. San Rafael (CA): Morgan & Claypool Life Sciences; 2011.

27. Eisenhofer G, Goldstein DS, Walther MM, Friberg P, Lenders JW, Keiser HR, Pacak K. Biochemical diagnosis of pheochromocytoma: How to distinguish true- from false-positive test results. *The Journal of clinical endocrinology and metabolism*. 2003;88:2656-2666

28. Sumner LW, Amberg A, Barrett D, Beale MH, Beger R, Daykin CA, Fan TW, Fiehn O, Goodacre R, Griffin JL, et al. Proposed minimum reporting standards for chemical analysis chemical analysis working group (CAWG) metabolomics standards initiative (MSI). *Metabolomics : Official journal of the Metabolomic Society*. 2007;3:211-221

**GRAPHIC ABSTRACT CREDITS**

The nephron figure used in the Graphical abstract was modified from the file ‘Physiology_of_Nephron’ (<https://commons.wikimedia.org/wiki/File:Physiology_of_Nephron.png>) by Madhero88, licensed under the Creative Commons Attribution 3.0 Unported license.

**STROBE Statement—Checklist**

|  | Item No | Recommendation | Page No |
| --- | --- | --- | --- |
| **Title and abstract** | 1 | (*a*) Indicate the study’s design with a commonly used term in the title or the abstract | 2 |
|  |  | (*b*) Provide in the abstract an informative and balanced summary of what was done and what was found | 2 |
| Introduction | | | |
| Background/rationale | 2 | Explain the scientific background and rationale for the investigation being reported | 3 |
| Objectives | 3 | State specific objectives, including any prespecified hypotheses | 3 |
| Methods | | | |
| Study design | 4 | Present key elements of study design early in the paper | 4 |
| Setting | 5 | Describe the setting, locations, and relevant dates, including periods of recruitment, exposure, follow-up, and data collection | 4 |
| Participants | 6 | (*a*) Give the eligibility criteria, and the sources and methods of selection of participants | 4 |
| Variables | 7 | Clearly define all outcomes, exposures, predictors, potential confounders, and effect modifiers. Give diagnostic criteria, if applicable | 4-6 |
| Data sources/ measurement | 8* | For each variable of interest, give sources of data and details of methods of assessment (measurement). Describe comparability of assessment methods if there is more than one group | 4-6;  Supp 3, 6-9 |
| Bias | 9 | Describe any efforts to address potential sources of bias | 4 |
| Study size | 10 | Explain how the study size was arrived at | 4 |
| Quantitative variables | 11 | Explain how quantitative variables were handled in the analyses. If applicable, describe which groupings were chosen and why | 6-7 |
| Statistical methods | 12 | (*a*) Describe all statistical methods, including those used to control for confounding | 6-7 |
|  |  | (*b*) Describe any methods used to examine subgroups and interactions | 6-7 |
|  |  | (*c*) Explain how missing data were addressed | 6 |
|  |  | (*d*) If applicable, describe analytical methods taking account of sampling strategy | 6-7 |
|  |  | (*e*) Describe any sensitivity analyses | n.a. |
| Results | | | |
| Participants | 13* | (a) Report numbers of individuals at each stage of study—eg numbers potentially eligible, examined for eligibility, confirmed eligible, included in the study, completing follow-up, and analysed | 8 |
|  |  | (b) Give reasons for non-participation at each stage | n.a |
|  |  | (c) Consider use of a flow diagram | n.a. |
| Descriptive data | 14* | (a) Give characteristics of study participants (eg demographic, clinical, social) and information on exposures and potential confounders | 8 |
|  |  | (b) Indicate number of participants with missing data for each variable of interest | 8-11  Tab 1-2 |
| Outcome data | 15* | Report numbers of outcome events or summary measures | 8-11 |
| Main results | 16 | (*a*) Give unadjusted estimates and, if applicable, confounder-adjusted estimates and their precision (eg, 95% confidence interval). Make clear which confounders were adjusted for and why they were included | 8-11  Tab 1-2 |
|  |  | (*b*) Report category boundaries when continuous variables were categorized | 8 |
|  |  | (*c*) If relevant, consider translating estimates of relative risk into absolute risk for a meaningful time period | n.a. |
| Other analyses | 17 | Report other analyses done—eg analyses of subgroups and interactions, and sensitivity analyses | 9-11 |
| Discussion | | | |
| Key results | 18 | Summarise key results with reference to study objectives | 12-16 |
| Limitations | 19 | Discuss limitations of the study, taking into account sources of potential bias or imprecision. Discuss both direction and magnitude of any potential bias | 14-16 |
| Interpretation | 20 | Give a cautious overall interpretation of results considering objectives, limitations, multiplicity of analyses, results from similar studies, and other relevant evidence | 15-16 |
| Generalisability | 21 | Discuss the generalisability (external validity) of the study results | 15-16 |
| Other information | | | |
| Funding | 22 | Give the source of funding and the role of the funders for the present study and, if applicable, for the original study on which the present article is based | 17 |
